# Nonlinear Harmonics: A Gateway to Enhanced Image Contrast and Material Discrimination

**DOI:** 10.1002/advs.202411556

**Published:** 2025-01-28

**Authors:** Pardis Biglarbeigi, Gourav Bhattacharya, Dewar Finlay, Amir Farokh Payam

**Affiliations:** ^1^ Department of Pharmacology & Therapeutics University of Liverpool Whelan Building, Liverpool England L69 3GE UK; ^2^ School of Engineering Ulster University York Street Belfast Northern Ireland BT15 1AP UK

**Keywords:** AFM, image contrast enhancement, image fusion, nonlinear harmonics

## Abstract

Recent advancements in atomic force microscopy (AFM) have enabled detailed exploration of materials at the molecular and atomic levels. These developments, however, pose a challenge: the data generated by microscopic and spectroscopic experiments are increasing rapidly in both size and complexity. Extracting meaningful physical insights from these datasets is challenging, particularly for multilayer heterogeneous nanoscale structures. In this paper, an unsupervised approach is presented to enhance AFM image contrast by analyzing the nonlinear response of a cantilever interacting with a material's surface using a wavelet‐based AFM. This method simultaneously measures different frequencies and harmonics in a single scan, without the need for additional hardware and exciting multiple cantilevers' eigenmodes. This developed AFM image contrast enhancement (AFM‐ICE) approach employs unsupervised learning, image processing, and image fusion techniques. The method is applied to interpret complex multilayer structures consist of defects, deposited nanoparticles and heterogeneities. Its substantial capability is demonstrated to improve image contrast and differentiate between various components. This methodology can pave the way for rapid and precise determination of material properties with enhanced resolution.

## Graphical abstract

An unsupervised AFM image contrast enhancement (AFM‐ICE) technique based on the integration of image fusion and histogram equalization, employing wavelet‐based analysis of nonlinear cantilever responses, enables improved visualization of complex multilayer structures with distinct defects, nanoparticles, and heterogeneities, all without additional hardware. This method enhances contrast and component differentiation, overcome artifacts due to the feedback error, facilitating precise material imaging.

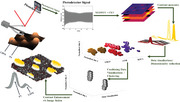

## 1 Main

Image enhancement aims to improve the dynamic range of an image, enhancing its visual effects and facilitating information extraction by human observers or computer systems. Advances in image and signal processing have made these techniques invaluable across diverse applications, including medical imaging,^[^ ^1^, ^2^ ^]^ image forensics,^[^ ^3^, ^4^ ^]^ satellite imaging,^[^ ^5^, ^6^ ^]^ nanomaterials, and biological observations.^[^ ^7^, ^8^ ^]^

Given the critical role of image enhancement, numerous studies have developed techniques to retain and enhance embedded information. These techniques fall into two broad categories: unsupervised and supervised algorithms.^[^ ^9^ ^]^ Common unsupervised techniques such as Histogram Equalization (HE) improves brightness distribution and contrast. Other examples of unsupervised techniques include Retinex theory, which models images as products of illumination and reflection,^[^ ^10^, ^11^ ^]^ and visual cortex neural networks, which simulate the mammalian visual cortex system.

Recently, Deep Learning (DL) methods, particularly Convolutional Neural Networks (CNN), have gained prominence in supervised image enhancement, notably in medical,^[^ ^12^, ^13^ ^]^ and ex‐vivo tissue imaging.^[^ ^14^ ^]^

Despite these advancements, existing techniques face limitations such as over‐enhancement, intensity shift, intensity saturation,^[^ ^15^, ^16^ ^]^ high computational cost, and the need for large training dataset. Consequently, image fusion techniques have been proposed^[^ ^15^ ^]^ to combine properties from multiple image derivatives, enhancing contrast and visual information. Fusion‐based techniques are aimed at incorporating the useful properties obtained from multiple derivatives of the original image to obtain an image with better contrast and visual information. Fusion techniques, combined with traditional methods like HE, have shown superior performance in medical image contrast enhancement.^[^ ^17^, ^18^ ^]^

In material sciences, particularly in nanoscale studies using microscopy, image contrast enhancement remains crucial yet underexplored. Among nanoscience microscopy techniques, atomic force microscopy (AFM) stands out for its ease of use, adaptability to ambient conditions, and straightforward sample preparation.^[^ ^19^ ^]^ AFM's versatility in imaging, characterizing, and manipulating materials beyond the molecular and atomic levels, provide valuable insights into nanoscale topography, material properties, and interactions.^[^ ^20^, ^21^ ^]^

The distinctive feature of AFM fields lies in the production of a substantial amount of high‐veracity experimental data presented as images. Although it offers prompt visualization of atomic structures, the analysis of this data has conventionally been confined to qualitative aspects. This includes emphasizing characteristics like the identification of structural and topological defects, interfaces, and so forth.^[^ ^22^, ^23^ ^]^

Over the last decade, various methodologies have emerged to quantify the AFM data. In these methodologies, atomic coordinates derived from image analysis are mapped onto a (postulated) mesoscopic order parameter field, offering insights into the surface properties. The typical procedure for such analyses involves the identification and fine‐tuning of nanoparticle positions, followed by the conversion of the measured coordinates into tangible physical quantities.^[^ ^24^, ^25^ ^]^

However, owing to the nanoscale composition of materials and the existence of noise, discerning materials, particularly in the context of nanoparticles, 2D and multilayer heterogeneous materials, pose a formidable challenge. Furthermore, the quest for minimizing noise and subsequently enhancing image contrast is a consistent pursuit across various microscopy techniques, including AFM. Recently, the application of machine learning and artificial intelligence techniques has been adopted to mitigate noise, extract material properties, and augment the image contrast in AFM.^[^ ^22^, ^23^, ^26^, ^27^, ^28^, ^29^ ^]^

On the other hand, in AFM, harmonic signals refer to higher‐order oscillations of the cantilever that arise from nonlinear interactions between the AFM tip and the sample surface. Unlike standard topography, which maps surface height, or phase imaging, which reflects energy dissipation and stiffness,^[^ ^30^, ^31^ ^]^ harmonic signals encapsulate a wealth of material properties at the nanoscale, including adhesion, elasticity, and viscoelasticity.^[^ ^32^ ^]^ When the tip contacts the sample, it experiences forces that are not purely elastic, leading to complex oscillatory responses where each harmonic reflects different facets of the interaction. By analyzing these harmonic components, it can be possible to distinguish mechanical properties across a sample and map variations in stiffness, adhesion, or viscoelasticity, transforming AFM from a simple topographical tool into a powerful instrument for probing mechanical and compositional contrasts at the nanoscale.^[^ ^33^, ^34^, ^35^ ^]^ Advanced AFM techniques like wavelet analysis^[^ ^36^ ^]^ and multifrequency AFM^[^ ^35^ ^]^ further exploit these harmonic signals to provide high‐resolution, spatially sensitive material maps, driving advancements in materials science, biology, and nanotechnology.^[^ ^35^, ^37^, ^38^, ^39^ ^]^

In this context, based on the integration of image fusion, histogram equalization, and the incorporation of embedded frequencies and harmonics data within the measured cantilever signal, we introduce an unsupervised algorithm named “AFM Image Contrast Enhancement (AFM‐ICE)”. This algorithm aims to enhance image contrast and effectively isolate defects and artefacts, facilitating the differentiation of various materials within complex, heterogeneous multilayer nanoscale structures. We demonstrate quantitative improvements in material contrast using the collective multi‐harmonics response. Employing our recently developed Wavelet Transform (WT)‐based AFM,^[^ ^36^ ^]^ we concurrently capture all frequency components embedded in the cantilever's response within the time needed to record a single pixel, eliminating the need for additional Lock‐in amplifiers (LIAs) or expand the number of eigenmodes.

We apply our method to study single wall carbon nanotube (SWCNT) deposited onto a substrate featuring a native oxide layer, deposited gold (Au) nanoparticles on multilayer highly oriented pyrolytic graphite (HOPG), as well as deposited multilayer graphene oxide (GO) on HOPG, and deposited gold nanoparticles on multilayer GO‐HOPG surfaces. These scenarios present challenges in distinguishing between layers and addressing the impact of feedback artefact and defects, aiming to accurately capture dimensional structure as well as discriminate between gold nanoparticles, GO, and HOPG within the images and determining the boundaries, defects and layers.

## 2 Results

**Figure 1:**
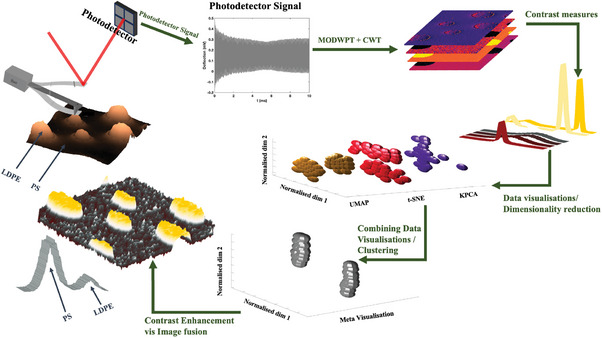
AFM Image contrast enhancement. Schematic of the proposed contrast enhancement of the AFM images with consideration of nonlinear harmonics arising from material properties, highlighting the experimental and numerical techniques used: high‐speed data acquisition of the photodetector signal, extraction of higher harmonics using MODWPT+CWT method, calculation of the normalized harmonic amplitudes through mixing image products at driven and non‐driven frequencies and their contrast measures, various data visualizations of the contrast measures, spectral analysis of combining the visualizations,^[^ ^40^ ^]^ followed by clustering, interpreting the clusters and associated material properties, using image fusion for contrast enhancement.

The diagram illustrating our WT‐based AFM method in combination with the proposed AFM‐ICE method is depicted in **Figure** **1**. Initially, dynamic‐mode AFM experiments were performed, where the cantilever was excited at its first resonance frequency, resulting in the monitoring of the signals continuously. Further, Wavelet‐based techniques were employed to extract the time‐frequency information of the time‐domain signal (see Materials and Methods and Section S1, Supporting Information). The resulting amplitude of the signal at the cantilever excitation frequency *f*_1_, second eigenfrequency *f*_2_, the second to sixth harmonics as well as a set of mixing products of all (details in Section S2, Supporting Information), are used to reconstruct the images. The images obtained from WT‐AFM exhibit high resolution, corresponding to the sampling frequency of the data acquisition board (DAQ) and have been proven to effectively represent the details of the sample's dynamic including transient behaviors up to microseconds (µ*s*) (Section S3, Figures S1–S4, Supporting Information).^[^ ^39^ ^]^

For this study, all images (*n* = 30) were downscaled by averaging to 256 × 256‐pixel images to facilitate better comparison with topography and phase images obtained from a lock‐in amplifier. Various image contrast measures and the spatial frequencies, as described in *Image Contrast Measures* section, were calculated for the images. Given the range of contrast measures and spectral frequencies considered for each image and the complex material characteristics of the samples, commonly used visualization methods‐ such as kernel Principal Component Analysis (kPCA), t‐distributed Stochastic Neighbor Embedding (t‐sne), and Uniform Manifold Approximation and Projection (UMAP)‐ have not proven to be reliable for embedding this dataset. Therefore, we adopted a spectral methodology, proposed by Ma, R. et al.^[^ ^40^ ^]^, which combines the results of different visualizations through weighted average of their normalized distance. In this study, we employed 8 scenarios of parameter tuning for kPCA (using Gaussian and Laplacian kernel), t‐sne and UMAP (using Euclidean, Cosine distance and Correlation) and performed the proposed spectral method (detailed in Section S4, Supporting Information). This method resulted in a meta‐visualization categorizing the images into two clusters: effective and non‐effective. Images in effective clusters displayed distinct details of the material characteristics. By fusing two of the normalized images in the effective cluster, we enhanced the raw images to represent more quantifiable information of the sample.

In order to examine the efficiency of the proposed methodology, we investigated four samples (i.e., I. SWCNT deposited onto a silicon substrate featuring a native oxide layer, II. multi‐layer GO‐HOPG, III. deposited Au‐ nanoparticles on multi‐layer HOPG, IV. deposited Au‐ nanoparticles on multi‐layer GO‐HOPG). In order to verify the presence of all these constituents and to examine the accuracy of our measurements we have carried out a detail Field Emission Scanning Electron Microscopy (FESEM)‐Energy‐Dispersive X‐ray Spectroscopy (EDX) analysis and the results are provided in Figures S5– S9 Section S5, Supporting Information.

**Figure 2:**
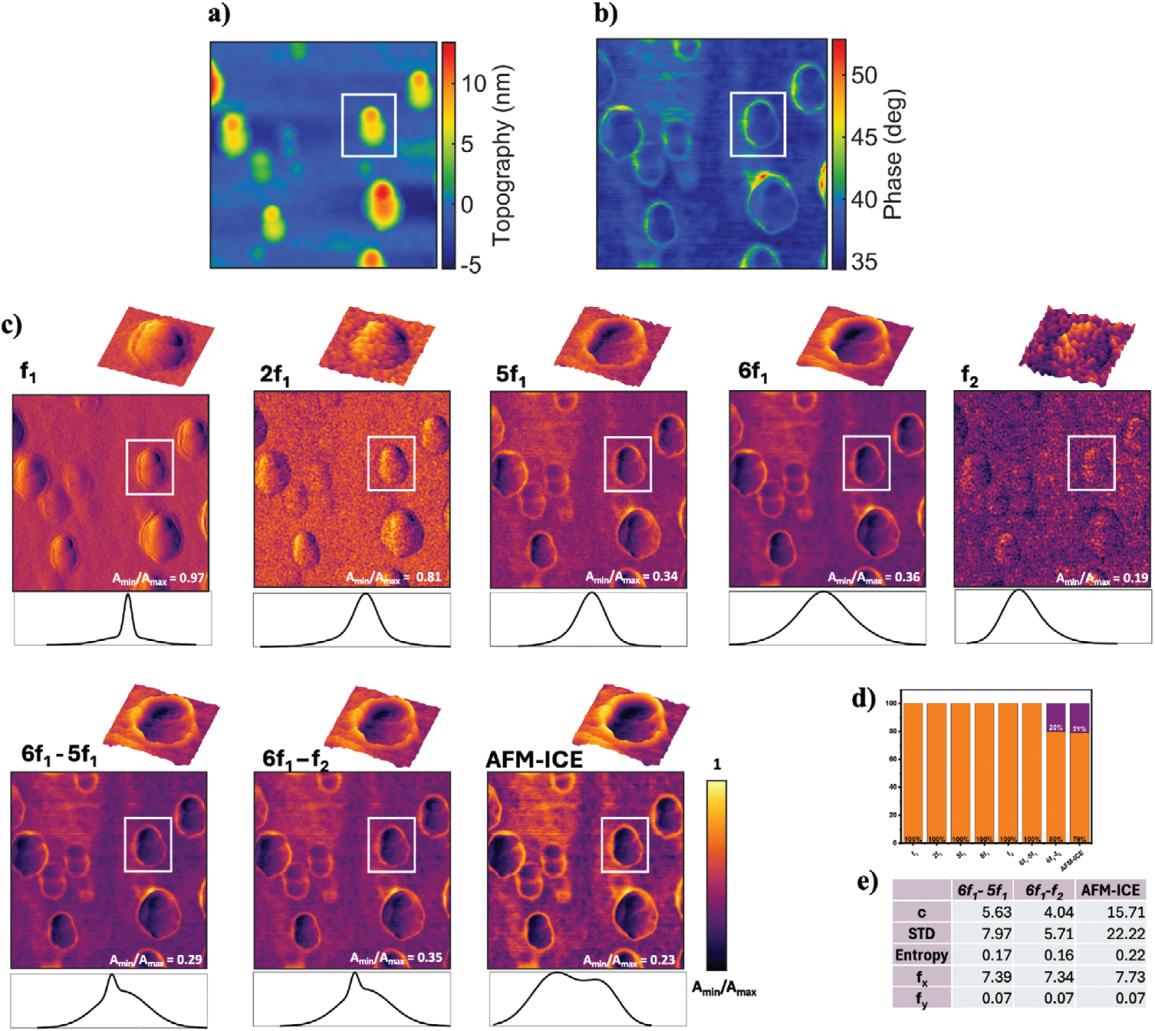
SWCNT sample. An area of 0.34 × 0.34 µm of the sample is investigated. The a) topography and b) phase images of the sample are obtained via LIA. c) The harmonic figures, further represent the normalised amplitude obtained from WT‐AFM method. A region of interest (ROI), shown with a box, of the sample is chosen to represent more details of the amplitude histogram of the materials with the zoomed‐in 3D figure of the ROI representing the amplitude in both color and z‐axis. d) Represents the calculated PDFs quantifying the material presence percentages in ROI. The orange colour is represented the SWCNT while the purple represents the substrate e) Represents the contrast measures and spatial frequency for the ROI of the selected images from the effective cluster as well as the image obtained from the proposed methodology.

**Figure 3:**
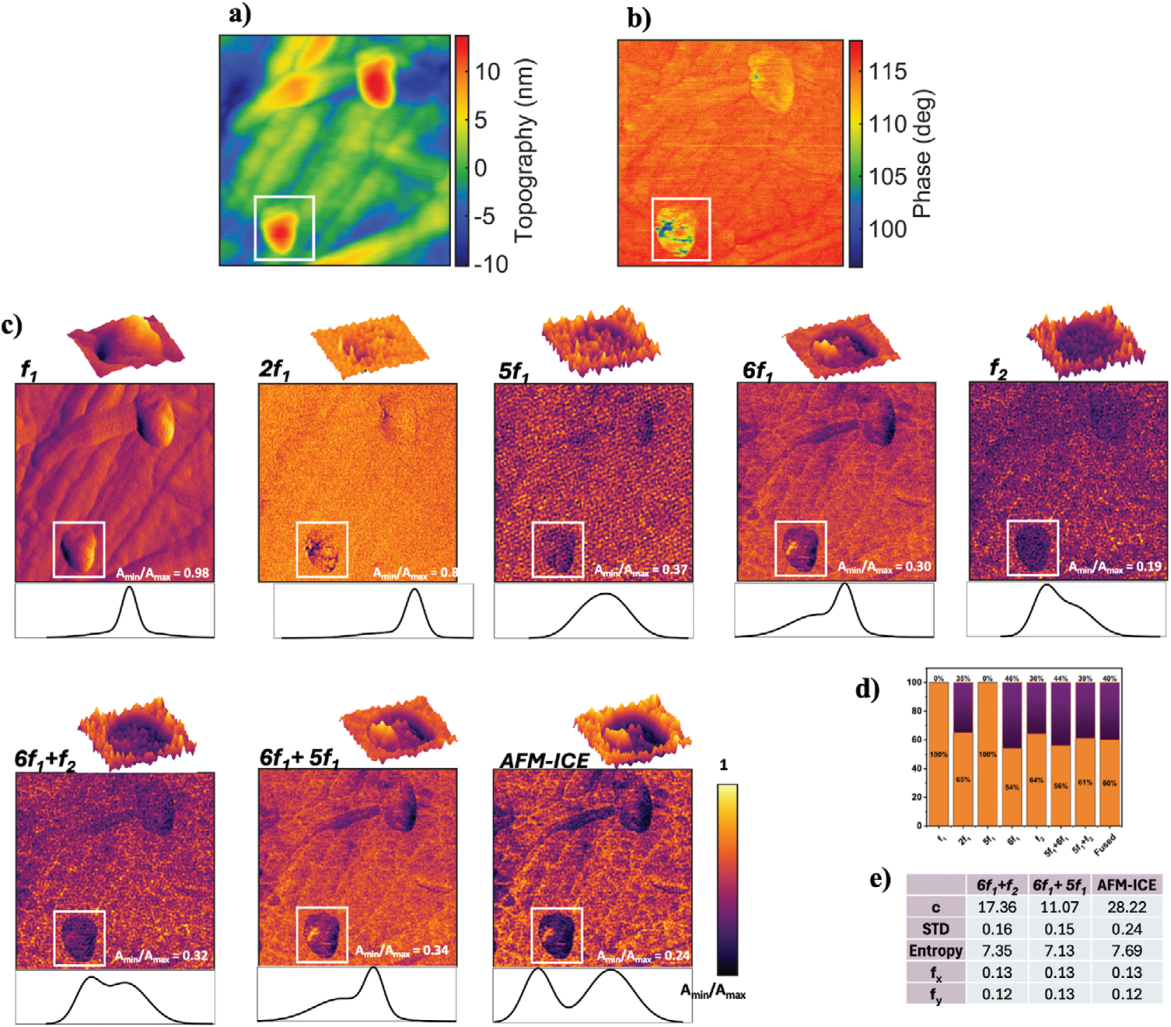
GO‐HOPG sample. An area of 0.78 × 0.78 µm of the sample is investigated. The a) topography and b) phase images of the sample are obtained via LIA. c) The harmonic figures, further represent the normalized amplitude obtained from WT‐AFM method. A region of interest (ROI), shown with a box, of the sample is chosen to represent more details of the amplitude histogram of the materials with the zoomed‐in 3D figure of the ROI representing the amplitude in both color and z‐axis. d) Represents the calculated PDFs quantifying the material presence percentages in ROI. The orange color represents GO while purple color represents HOPG. e) Represents the contrast measures and spatial frequency for the ROI of the selected images from the effective cluster as well as the image obtained from the proposed methodology.

**Figure 4:**
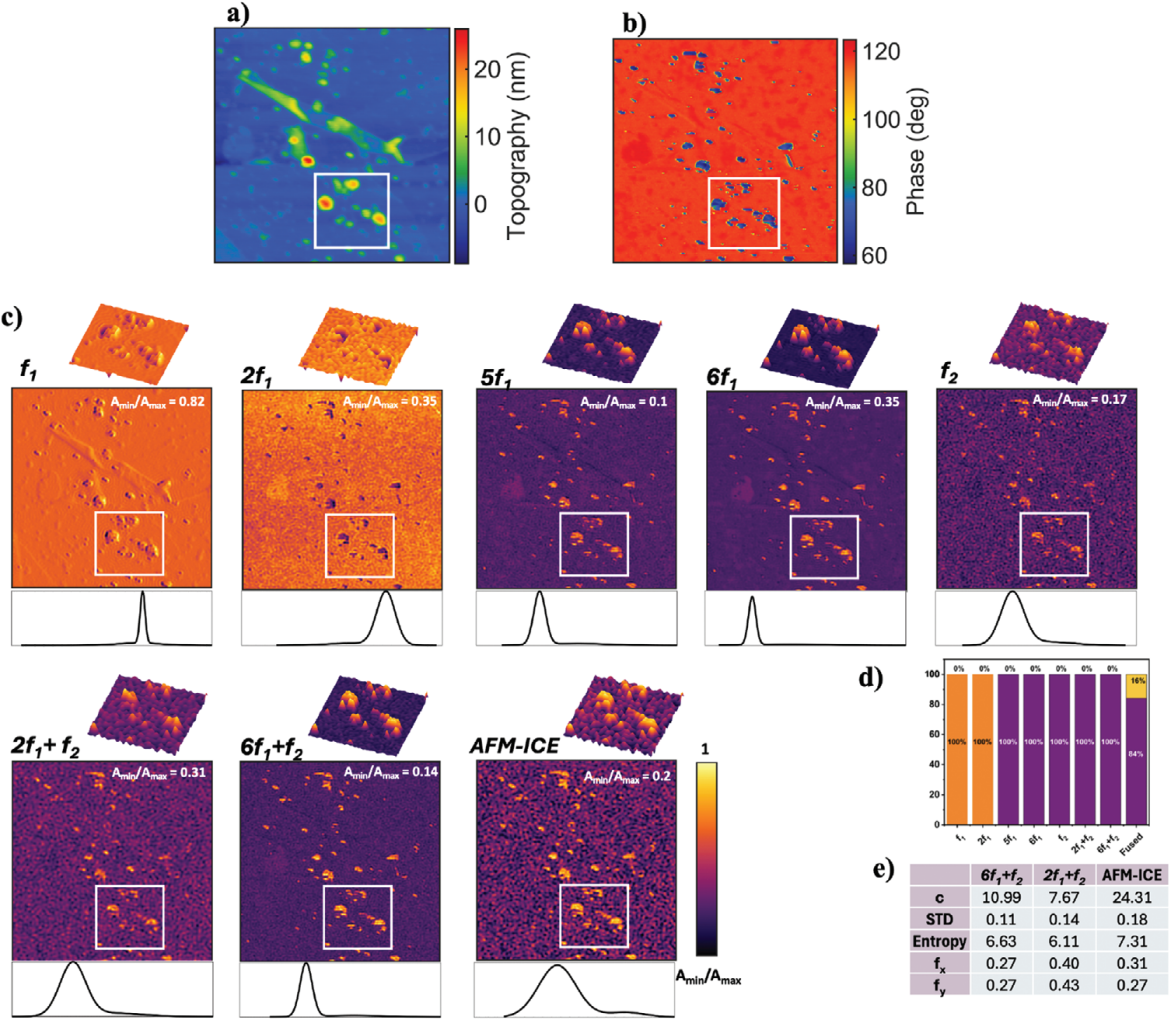
Deposited Au‐nanoparticles on multi‐layer HOPG sample. An area of 2 × 2 µm of the sample is investigated. The a) topography and b) phase images of the sample are obtained via LIA. c) The harmonic figures, further represent the normalized amplitude obtained from WT‐AFM method. A region of interest (ROI), shown with a box, of the sample is chosen to represent more details of the amplitude histogram of the materials with the zoomed‐in 3D figure of the ROI representing the amplitude in both color and z‐axis. d) Represents the calculated PDFs quantifying the material presence percentages in ROI. The purple color represents HOPG while the orange represents gold NPs. e) Represents the contrast measures and spatial frequency for the ROI of the selected images from the effective cluster as well as the image obtained from the proposed methodology.

**Figure 5:**
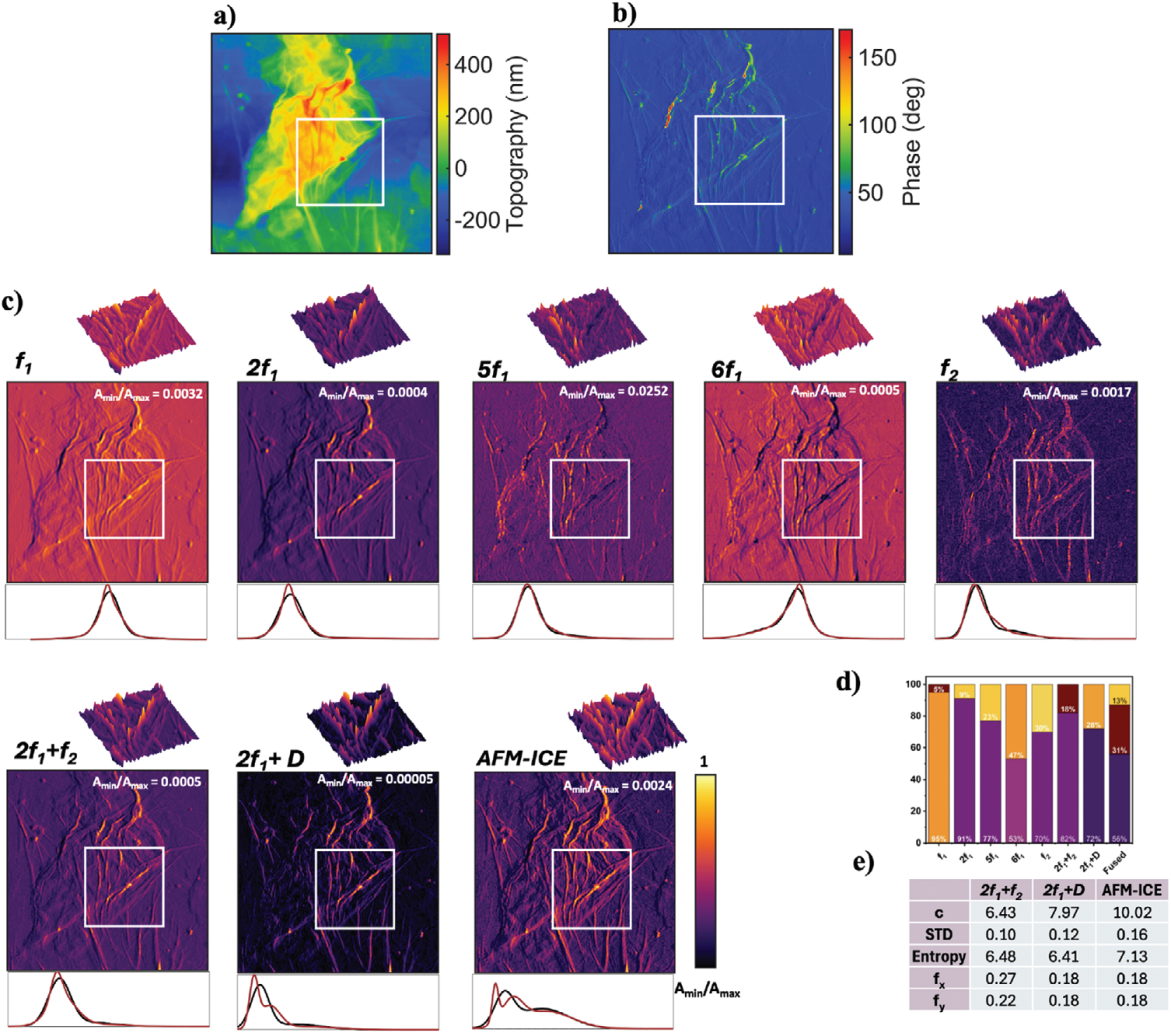
Deposited Au‐nanoparticles on multi‐layer GO‐HOPG sample. An area of 12 × 12 µm of the sample is investigated. The a) topography and b) phase images of the sample are obtained via LIA. c) The harmonic figures, further represent normalized amplitude obtained from WT‐AFM method. A region of interest (ROI), shown with a box, of the sample is chosen to represent more details of the amplitude histogram of the materials with the zoomed‐in 3D figure of the ROI representing the amplitude in both color and z‐axis. The histogram shown in BLACK represents the summation of two fitted normalized histograms. The histogram shown in RED represents the summation of three fitted normalized histograms. d) Represents the calculated PDFs quantifying the material presence percentages. The orange represents HOPG, purple Go and brown gold NPs. e) Represents the contrast measures and spatial frequency for the selected images from the effective cluster as well as the image obtained from the proposed methodology.

I. **SWCNTs**: This sample is consisting of SWCNTs deposited onto a silicon substrate featuring a native oxide layer (See Figure S5, for its FESEM‐EDX analysis, Supporting Information). For validation of our approach SWCNTs are ideal because they have both material contrast and a clearly defined 3D structure, including tube width, length and height, that can be measured. By using such standards, we could demonstrate that our method not only improves contrast but also accurately captures the physical dimensions and features of the sample in the case that the tip is sharper than the sample features and overcoming the artifacts in the topography image due to the feedback error, thereby validating the method's utility for precise structural analysis. The representative topography and phase images are given in **Figure** **2**. For our measurements, SWCNTs were dispersed in isopropanol (IPA) and deposited onto a silicon substrate. Stacking, partial bundling, and minor agglomeration of the nanotubes were observed, attributed to the strong van der Waals forces between SWCNTs, which persist even after dispersion and promote re‐aggregation. Sonication in IPA for one hour effectively disrupted larger bundles but was insufficient to fully overcome the intrinsic π − π interactions driving nanotube cohesion. During deposition, capillary forces generated by solvent evaporation further exacerbated bundling, as nanotubes were drawn together during drying. The silicon substrate also influenced these interactions, with its moderate surface energy facilitating adhesion and partial alignment of the nanotubes, contributing to the observed stacking and aggregation.
  The reconstructed images of the excited frequency *f*_1_, second eigenfrequency *f*_2_, and the 2*f*_1_, 5*f*_1_, and 6*f*_1_ harmonics are presented in Figure 2 and Figure S1, Supporting Information. As shown, the presence of two materials in the region of interest (ROI) is qualitatively visible; however, due to the minimal percentage of substrate in this area, the histograms predominantly display a single peak. In contrast, the AFM‐ICE image histogram reveals two distinct peaks, highlighting the superior performance of our technique in distinguishing between materials. Furthermore, by fusing the mixing product of the harmonics, selected from the effective cluster (details in Section S6 Table S3, Supporting Information), using our proposed AFM‐ICE technique, we were able to increase the contrast of the sample image, as the fused image shows the highest value for sample contrast (c), standard deviation (STD), and Entropy. The details of these image contrast measures are given in *Image Contrast Measures* section. Furthermore, by applying histogram‐based probability density function (PDF) determination approach (more details in *Histogram‐Based Probability Distribution Function (PDF) Determination* section), we could quantify the percentage of SWCNT and substrate appeared in the images. As seen, just in the combined sixth harmonic and second eigenmode image which is used for fusion and fused image the PDF quantify the percentage of two materials existing in the ROI. Furthermore, as shown in Figure S10 (Section S7, Supporting Information), the dimensional structure of SWCNT in the AFM‐ICE image not only can be captured accurately but also can overcome the topography artifacts due to the feedback error. As can be seen from comparison between topography and phase images of SWCNTs in Figure 2 as well as Figure S10, Supporting Information, there is a difference between width and length of SWCNTs. This arises from feedback error of AFM measurements. The topography is recorded by scanning the sample and keeping the cantilever's oscillation amplitude at the setpoint value by means of a feedback loop. In fact, a change in tip‐sample distance yields a change in interaction force and hence frequency, amplitude and phase. By monitoring the value of the oscillation amplitude, the feedback loop detects changes in amplitude and accordingly lifts or lowers the probe holder over the surface in order to keep it constant. The probe holder movement is recorded and reflects the topography of the sample. Inherently the action of the feedback occurs at a time interval after the topography changes, both because of its finite response and the delay in change of the cantilever's oscillation. Such delay is the cause of error in recording the topography, which can be detected by monitoring the amplitude, and phase signals. As can be seen, the AFM‐ICE image not only clearly enhance the image contrast, where the SWCNTs are more visible than topography images, but also the length and width dimensions of SWCNTs can be measured more accurately than topography as our method overcomes the artifact of topography image arising from feedback error. Also, we have performed correlation analysis between topography with harmonics images and AFM‐ICE image, and as the results of Table S6, Supporting Information depicts, the highest correlation is obtained for AFM‐ICE which verifies that the improved contrast corresponds to actual, measurable features.
II. **GO‐HOPG**: This sample includes a deposited multilayer GO on HOPG. The hybrid structure combines HOPG's high conductivity with GO's tuneable chemistry, making it suitable for high‐performance sensors and electronic devices.^[^ ^41^, ^42^, ^43^, ^44^ ^]^ However, due to atomic steps of the HOPG, it is not possible to distinguish the GO and the HOPG from the topographic information alone,^[^ ^45^ ^]^ as seen in the topography image of **Figure** **3**.
  Image contrast and resolution enhancement, demonstrated in Figure 3 faciliate the study of nanoscale features, defects, and layer interactions of GO and HOPG. This is crucial for electronic, optical, and mechanical characterizations essential for applications in catalysis, energy storage, and advanced composites. The phase image in Figure 3 reveals that the measurement was performed in an intermittent contact regime, with phase exceeding 90°, generating harmonics.
  The reconstructed images of the excited frequency *f*_1_, second eigenfrequency *f*_2_, and the 2*f*_1_, 5*f*_1_, and 6*f*_1_ harmonics are presented in Figure 3 and Figure S2, Supporting Information. As can be seen, in 6*f*_1_ and *f*_2_ images, the presence of two materials in the ROI are apparent in the histograms. However, by fusing the mixing product of the harmonics, selected from the effective clusters (details in Section S6 Table S3, Supporting Information), using our proposed AFM‐ICE technique, we were able to increase the contrast of the sample image, as the fused image shows the highest value for sample contrast (*c*), standard deviation (*STD*), and *Entropy*. Furthermore, by applying histogram‐based PDF determination approach (more details in* Histogram‐Based Probability Distribution Function (PDF) Determination* section), we could quantify the percentage of GO and HOPG appeared in the images. From the figure, it is evident that for HOPG/GO, most images (excluding those obtained from *f*_1_ and 5*f*_1_) show two distinct peaks, one corresponding to HOPG and the other to GO. The histogram from *f*_1_ and 5*f*_1_ only extracted one material due to the presence of a single peak. In contrast, other histograms exhibited two peaks, allowing the PDF approach to quantify the presence percentages of both materials. The histogram of the fused sample, achieved by AFM‐ICE methodology, having the minimum histogram overlap, demonstrates the highest contrast, and clear distinction between GO and HOPG. The deconvolution of the peaks further enhances the accuracy of determining the presence percentage of GO and HOPG in the fused image.
III. **Deposited gold nanoparticles on multilayer HOPG**: Enhanced image contrast and precise characterization techniques are crucial for identifying and isolating gold nanoparticles deposited on HOPG substrate. These improvements provide a better understanding of the nanoparticles' distribution, interaction, and functional properties, essential for applications such as catalysis and sensor functionality.^[^ ^46^, ^47^, ^48^ ^]^
  The topography of this sample, shown in **Figure** **4**, illustrates the challenge of estimating the size and location of Au‐ nanoparticles. The harmonic images exhibit contrast reversal, indicating two distinct interaction regimes. In the phase images, certain regions show a phase less than 90°, signifying dominant repulsive interactions generating the harmonics. Other regions exhibit a phase greater than 90°, indicating intermittent contact between the cantilever and the surface. Additionally, certain features appear in the topography and *f*_1_ images but disappear in the phase and other harmonic images. Moreover, the harmonics of the sample show some information about the gold particles, however, due to the small size of the particles, as well as the low contrast of the images, the fitted normalized histogram is not able to show the intensity of the gold particles, and hence resulting in low values of the sample contrast, *c*. By applying the proposed AFM‐ICE methodology, the contrast of the image is significantly increased while the fitted normalized histogram can show two local peaks and therefore clarify the presence of two materials, i.e., Au‐ nanoparticles and HOPG on the sample. The higher spatial frequency of the fused image in the x‐direction, *f*_*x*_, compared to 6*f*_1_ + *f*_2_ illustrates the presence of abrupt changes in the fused image that corresponds to the representation of more edges and details in the fused image. On the contrary, *f*_*x*_ in the fused image is lower than that of 2*f*_1_ + *f*_2_, which shows that the fused images present more global features. Containing more details and more global features compared to the two initial images before fusion has resulted in enhanced contrast and consequently, higher values of *c*, *STD*, and Entropy for the fused image. Our image analysis also can explain the additional features in topography could be related to the contamination, defects as well as the agglomeration of hydrocarbons during the HOPG preparation. Our proposed method can clearly remove those effects from the final image. Also, while qualitatively the presence of Au‐ nanoparticles observed in all images, as the amount of Au is very minimal, it could not be detected through standard harmonic analysis. However, applying histogram‐based PDF determination on the images, demonstrates that the fused image distinctly differentiates and quantify the percentage of the two components and significantly enhances the image contrast.
IV. **Deposited Au‐nanoparticles on multilayer GO‐HOPG**: This sample consists of three materials of GO, HOPG, and deposited Au‐nanoparticles. This composite structure synergizes HOPG's exceptional electrical and mechanical properties,^[^ ^49^ ^]^ GO's tuneable surface chemistry,^[^ ^50^, ^51^ ^]^ and gold nanoparticles' unique optical and catalytic characteristics.^[^ ^52^, ^53^ ^]^ Enhancing the image contrast of this heterogeneous nanoscale structure, facilitate understanding gold nanoparticle distribution and interaction with the GO‐HOPG substrate and ensures the optimization of material and device performance crucial for catalysis, biosensing, and nanoelectronics applications.^[^ ^54^, ^55^, ^56^, ^57^, ^58^ ^]^ While the topography image consists of different contrast features, it is not possible to distinguish between three samples present on the image. Also, the phase images demonstrate two different interaction regimes in the measurements which is the cause of reverse contrast observed in the harmonics' images. While, as observed in **Figure** **5** and Figure S4, Supporting Information, the topography or any of the images corresponding to the harmonics of the sample show the distinction between the features, accordingly, the histograms of the ROI for *f*_1_, 2*f*_1_, 5*f*_1_, 6*f*_1_, and *f*_2_ represent only one peak which may lead to this understanding that there is only one material present in the sample. However, this is mainly due to the contrast of the images calculated from WT‐AFM. By applying the AFM‐ICE methodology on two of the images from the effective cluster (details in Section S6 Table S5, Supporting Information), we were able to enhance the image to show higher contrast with higher values of *c*, *STD*, and *Entropy*. For this sample, we further examined the methodology's ability to enhance image contrast and to represent the three materials in the ROI‐fitted histogram. Initially, we fitted a summation of two normal distributions to calculate *c*, as suggested by Forchheimer, D. et al.^[^ ^26^ ^]^ (shown with the black histogram in the subfigures of Figure 5). Subsequently, we fitted a summation of three normal distributions to the normalized histogram of the ROI, shown in red. The fused image uniquely exhibits three peaks corresponding to the intensity of the three materials present in the ROI. The decrease in spatial frequencies in both *x* and *y* direction for the fused image indicates the presence of more global information about the shapes, and orientation of the fused image. Similar to the Au‐HOPG sample, the tertiary configuration of Au‐HOPG‐GO shows that the minimal amount of Au remains undetected through standard harmonic analysis. However, the histogram‐based PDF determination, applied to the fused image clearly differentiates the components and proves that the proposed AFM‐ICE method could significantly enhance image contrast. This sample is chosen to represent more details of the amplitude histogram of the materials with the zoomed‐in 3D figure of the ROI representing the amplitude in both color and z‐axis. The histogram shown in BLACK represents the summation of two fitted normalized histograms. The histogram shown in RED represents the summation of three fitted normalized histograms.

## 3 Discussion

Our study demonstrates the capability and potential of the proposed AFM‐ICE methodology, which employs nonlinear harmonics to improve image contrast, overcome topography artifacts due to feedback error, and material discrimination in AFM. This technique addresses the complexity of modern microscopic datasets by utilizing a wavelet‐based AFM approach to simultaneously measure the phase and amplitude of different frequencies in a single scan. By integrating image processing, unsupervised learning algorithms, and image fusion techniques, AFM‐ICE enhances AFM images, enabling the clear distinction of various components within complex, heterogeneous nanoscale structures.

The WT‐based AFM technique generates high‐resolution images dependent on the DAQ's sampling frequency. However, to facilitate a better comparison with standard topography images, the images were scaled down to 256 × 256 pixels. This reduction process ensures computational efficiency and feasibility, even when dealing with larger datasets. The novelty of this method lies in its capacity to capture and utilize higher‐order harmonics and frequencies present in the cantilever signal to enhance image contrast, discriminate the components of the image, and remove artefacts and defects from the images, features typically overlooked in standard AFM imaging protocols. This capability significantly enhances image contrast, facilitating clearer differentiation between materials within complex, multilayer nanoscale structures. The AFM‐ICE methodology was tested on four sample scenarios: SWCNT‐Silicon substrate with native oxide layer, corresponding, GO‐HOPG, Au‐ nanoparticles deposited on multilayer HOPG, and Au‐ nanoparticles deposited on multilayer GO‐HOPG.

For SWCNTs, the method not only enhance the image contrast of SWCNTs but also, could remove the topography artifact due to the feedback error and capture the physical structure and dimensions of SWCNTs. For the GO‐HOPG sample, the method distinguished GO and HOPG layers and assisted in accurate quantification of the percentage of each in the fused image. In the Au‐ nanoparticles on multilayer HOPG sample, the approach facilitated precise identification and characterization of nanoparticle distribution. For the Au‐ nanoparticles on multilayer GO‐HOPG sample, AFM‐ICE successfully differentiated between the three materials, a challenging task using conventional image analysis techniques.

Furthermore, we have applied our approach, on different scan areas and regions of interest of the samples to validate the comprehensiveness and robustness of our AFM‐ICE platform. Our results demonstrated in Section S8, Supporting Information, shows statistically significant improvement in image contrast and materials discrimination for all the heterogeneous materials under study.

Our results highlight the effectiveness of the histogram‐based PDF determination approach in quantifying material composition within images. While qualitative analysis of images often shows different components, the histogram peaks of associated images sometimes fail to distinguish these components due to low contrast or overlapping peaks. The AFM‐ICE methodology enhances image contrast, allowing for the deconvolution of histogram peaks. This deconvolution provides a clearer representation of the components present, enabling the precise determination of their percentages. Enhanced contrast through image fusion resulted in images with higher values of sample contrast, standard deviation, and entropy. The process of integrating multiple visualization methods, such as kPCA, t‐SNE, and UMAP, with various parameter tunings, is necessary to achieve reliable image contrast enhancement but may require considerable computational resources, particularly for analyzing larger images obtained from WT‐AFM while investigating material dynamics. To address these computational demands, future optimizations could involve parallel processing techniques, use of High‐Performance Computing (HPC) resources and more efficient algorithms for spectral analysis. Additionally, employing advanced machine learning techniques may further streamline the process, reducing computation time while maintaining accuracy. Future research will focus on several key areas to further enhance the AFM‐ICE methodology. Exploring alternative spectral analysis methods and parameter tuning will improve the categorization of images and the resulting contrast enhancements. Incorporation of more sophisticated machine learning algorithms will automate the process of image enhancement and material differentiation.^[^ ^59^, ^60^ ^]^ These algorithms could include deep learning techniques,^[^ ^61^ ^]^ which may offer improved accuracy and efficiency. Development of adaptive algorithms capable of dynamically adjusting parameters based on the specific characteristics of each dataset will enhance the robustness and applicability of the methodology. Furthermore, while the main applicability and advantage of the AFM‐ICE method is for materials discrimination in heterogeneous samples, combining the AFM‐ICE with deep learning methodologies such as CNN^[^ ^61^ ^]^ could extend the capability of the image fusion technique to enhance the tip deconvolution approach for the case that the sample is sharper and smaller than tip radius. Extending the application of the AFM‐ICE methodology to other types of AFM imaging, such as Kelvin Probe Force Microscopy (KPFM), magnetic force microscopy, and conductive AFM, will demonstrate the versatility and broad applicability of the methodology across different AFM techniques.

Furthermore, to retain physical interpretability in image fusion, a promising direction is to incorporate harmonic signals into a model‐based framework. In this context, there are physical models that use harmonics to calculate material properties such as Young's modulus and viscosity.^[^ ^33^, ^34^ ^]^

To address the potential for image fusion in mapping, while our current work focuses on enhancing contrast and material discrimination, there's an opportunity to integrate image fusion with physical models. This approach can employ known relationships between harmonic responses and material properties, allowing researchers to map specific attributes like adhesion or viscoelasticity rather than simply enhancing contrast. A model‐based fusion method would preserve the mechanical insights embedded in harmonic signals, enhancing the fused images' value for both qualitative and quantitative analysis of complex materials. Additionally, recent advancements, such as the methodology by Petrov et al., ^[^ ^62^ ^]^, show potential for using machine learning (ML) in material identification and in correlating nanostructures with macroscopic properties. Integrating image fusion with a model‐based framework and ML could further improve material property mapping.

To summarize, AFM‐ICE significantly enhances image contrast and material discrimination in AFM. The use of higher harmonics and mixing products reconstructed high‐resolution images, providing detailed insights into the sample's dynamic behavior. The histogram‐based PDF quantified material composition, demonstrating the method's precision. Looking ahead, future advancements could explore refining the AFM‐ICE methodology to handle larger datasets and incorporating more sophisticated algorithms, broadening its applicability in studying complex heterostructures, nanoscale phenomena, and interfacial charge kinetics. The promising results from this study suggest that AFM‐ICE can become an invaluable tool in material imaging and characterization, driving progress in various scientific and industrial domains.

## 4 Materials and Methods

### 4.1 Materials

Tetrachloroauric (III) acid trihydrate (*HAuCl*_4_.3*H*_2_ *O*), Sodium hydroxide (*NaOH*), and acetic acid of analytical reagent (AR) grade were procured from Sigma Aldrich and utilized without further purification. Highly Oriented Pyrolytic Graphite (HOPG) with a mosaic spread value of 3.5° ± 1.5° was acquired from µmasch (MikroMasch Europe, operated by NanoAndMore GmbH). Graphene oxide (GO) powder of AR grade was obtained from Sigma–Aldrich (Merck, UK). Single‐wall carbon nanotube (SWCNT) flakes were purchased from Ossila (UK). Prior to experimentation, the HOPG surface underwent cleavage using Scotch Magic Tape (3M, USA). Ultrapure deionized (DI) water from a Millipore Milli‐Q system, with an electrical resistivity of 18.2 *M*Ω cm, was utilized for the preparation of aqueous solutions.

### 4.2 Preparation of AuNP‐Decorated HOPG

Gold nanoparticles were synthesized from *HAuCl*_4_ using a citrate‐mediated thermal reduction method. Specifically, 1 mL of a 0.175*M* aqueous citric acid solution was added to a solution of 0.52 *mM* *HAuCl*_4_ and stirred continuously. After 12 s, the pH of the solution was adjusted by adding 1*M* aqueous *NaOH* solution, followed by cooling. The resulting gold nano‐dispersion was collected and stored for further use. A 1 *mM* aqueous dispersion containing gold nanoparticles (with an average particle size of approximately 40*nm*) was prepared. Subsequently, 20 µL of the as‐prepared nanodispersion were drop‐cast onto the freshly cleaved HOPG sample. The resulting assembly was air‐dried at room temperature, yielding the HOPG specimen decorated with gold (Au) for subsequent AFM measurement.

### 4.3 Preparation of GO‐Decorated HOPG

Graphene oxide (GO) powder was obtained from Sigma–Aldrich (Merck, UK), and a water‐based dispersion with a concentration of 0.1 mg mL^−1^ was prepared using ultrapure DI water with a resistivity of 18*M*Ω cm (Millipore Milli‐Q system, USA). Subsequently, the dispersion was drop‐cast onto a freshly cleaved HOPG surface and allowed to dry under ambient conditions.

### 4.4 Preparation of AuNP Decorated GO‐HOPG

To prepare the AuNP‐decorated GO‐modified HOPG sample, 20 µL of 1 *mM* aqueous dispersion of AuNPs were subsequently drop cast on the as‐obtained GO decorated HOPG surface and dried under ambient condition.

### 4.5 Preparation of SWCNT‐Decorated Silicon

The as‐purchased flakes of SWCNT were dispersed in Isopropyl alcohol and sonicated for an hour. A 1 mg mL^−1^ dispersion was then dropped over a clean silicon substrate, dried in an ambient atmosphere, and used for AFM measurement.

### 4.6 Microscopy Measurements—FESEM‐EDX

The morphological features and elemental composition of the samples were analyzed using a HITACHI SU5000 scanning electron microscope (SEM) equipped with an energy‐dispersive X‐ray spectroscopy (EDX) detector. SEM imaging and EDX mapping were performed at an acceleration voltage of 10*kV* and a working distance of approximately 6 *mm*.

### 4.7 Microscopy Measurements—AFM

All samples were analyzed using the Asylum Research Oxford Jupiter system, operating in tapping mode under ambient conditions. Imaging was conducted with an FMV‐A cantilever (Bruker) having dimensions of 225 µm in length, 30 µm in width, and 2.75 µm in thickness, with a spring constant of 1.7*Nm* ^−1^. The following parameters were used for imaging: first eigenfrequency (*f*_1_) of 68.392 *kHz*, optical lever sensitivity (InVOLS) of 129.57 *nm*/*V*, scan rate of 2.0032*Hz*, with a free amplitude of 1*V*, and set point of 0.45*V*. The spring constant was calibrated from the thermal noise spectrum using the Saeder method.^[^ ^63^ ^]^

### 4.8 Harmonic Detection Using WT

Wavelet‐based techniques are important mathematical tools in signal processing that are widely used to analyze stationary and time‐varying signals. Wavelets have the advantage of using varying window sizes, being wide windows for low‐frequency and narrow windows for high‐frequency components, to represent an ideal time‐frequency localization. Owing to the varying window sizes, wavelets are adapted to the transients at different frequency scales and therefore are pragmatic tools in detecting and visualizing non‐stationary harmonics of the signal.^[^ ^64^ ^]^

When applied to a signal, Wavelet Transform (WT) produces time‐frequency components of the signal for each instant. WT consists of wavelet and transform functions. Wavelet, or mother wavelet, is a small oscillatory wavelike function that can be dilated by changing its window size and translated by changing its location. The convolution of the mother wavelet and the signal results in wavelet transform. WT can be classified into two general categories of Continuous Wavelet Transform (CWT) and Discrete Wavelet Transform (DWT). CWT provides a complete representation of a signal by letting the translation and dilation parameters of the mother wavelet vary continuously, as opposed to DWT which calculates the coefficients at different decomposition levels. CWT is, therefore, considered to be able to detect the harmonics and sub‐harmonics of a signal.^[^ ^65^ ^]^ However, due to the deficiencies associated with the CWT filter banks, the amplitude obtained for the higher harmonics may show fluctuations.^[^ ^39^ ^]^ Furthermore, due to the logarithmic decomposition of frequency spectra and sub‐sampling of decomposition levels which results in time‐varying transformation, DWT cannot deliver an accurate representation of harmonics of the photodetector signal (more information in Section S1, Supplementary Information).^[^ ^66^ ^]^

To overcome these shortcomings, Walden, A.T. and Cristan, A.C.^[^ ^67^ ^]^ introduced Maximal Overlap Discrete Wavelet Packet Transform (MODWPT), which is defined as a time‐invariant transform by decomposing the entire signal using several band‐pass filters. At each level of decomposition, MODWPT provides equal frequency passbands and the decomposed coefficients at each frequency band is perfectly in line with the signal due to the application of zero‐phase filtering (mathematical description of MODWT can be found in Section S1, Supplementary Information).

In this paper, we decomposed the photodetector signal using MODWPT into 7 levels. The decomposition coefficients are calculated by using a high‐order Daubechies wavelet (*db*45) as the mother wavelet to ensure obtaining smooth and sinusoidal coefficients.^[^ ^39^ ^]^ The decomposed coefficients are then processed using CWT to obtain the amplitude of the signal at the cantilever excitation frequency *f*_1_,^[^ ^36^ ^]^, second eigenfrequency *f*_2_, and the second to sixth harmonic (i.e., 2*f*_1_, 3*f*_1_, 4*f*_1_, 5*f*_1_, 6*f*_1_) of the cantilever signal. However, it is worth noting that, since the experiments have been implemented in an air environment, 3*f*_1_ and 4*f*_1_ did not contain much information for any of the samples. Therefore, they have been eliminated from the analysis.

### 4.9 Image Contrast Measures

In order to better analyze the cantilever dynamics and map the material properties, we used the higher harmonic amplitude and phase images obtained by WT‐AFM and calculated four different image contrast measures, i.e., sample contrast index, image standard deviation, entropy as well as the images' spatial frequency. For this purpose, we obtained a combination of normalized harmonic amplitudes by mixing image products at driven and non‐driven frequencies to obtain more information, shown by Forchheimer, D. et al.^[^ ^26^ ^]^

- **Sample Contrast (*c*)** is calculated by considering the image histogram. For this purpose, the histograms of each of the images of harmonic amplitudes are initially normalized, x. Then a Gaussian Mixture Model (GMM) is fitted to the normalized histograms. GMM is a probabilistic model that represents the normally distributed sub‐materials within the overall sample.
  A summation of two normal distributions is widely used to fit the normalized histograms of samples that consist of two materials, as defined in Equation 1.
(1) $$f\left(x\right)=p\frac{1}{\sigma_{1}\sqrt{2\pi }}e^{-\frac{{\left(x-\mu_{1}\right)}^{2}}{2\sigma_{1}^{2}}}+\left(1-p\right)\frac{1}{\sigma_{2}\sqrt{2\pi }}e^{-\frac{{\left(x-\mu_{2}\right)}^{2}}{2\sigma_{2}^{2}}}$$
  where µ and σ^2^ denote the mean and variance of each of the two fitted normal distributions 1 and 2, *p* presents the ratio of the pixels in the first fitted distribution and 1 − *p* denotes the ratio of pixels presented in the second fitted distribution. Further, the contrast measure is calculated as defined in Equation 2. Higher values of *c* show higher contrast in the image.
(2) $$c=\sqrt{\frac{{\left(\mu_{1}-\mu_{2}\right)}^{2}}{\sigma_{1}^{2}+\sigma_{2}^{2}}}$$
  However, more investigation is required when a sample consists of more than two materials (e.g. deposited gold nanoparticles on a multilayer GO‐HOPG sample). In this study, we show that a Gaussian Mixture of more than two normal distributions can also provide more information on the image. However, this requires high contrast in the image (more information in Section S8, Supplementary Information).
- **Standard deviation (SD)** is used to represent the variability of the data compared to its average. In an image, the increase in SD shows a higher contrast. SD of an image can be calculated as Equation (3).
(3) $$\mathrm{SD}=\frac{\sqrt{\sum_{i=1}^{X}\sum_{j=1}^{Y}|F\left(i,j\right)-\mu {|}^{2}}}{\mathrm{XY}}$$
  where *X* and *Y* represent the dimensions, *F*(*i*, *j*) represents the pixel value located in (*i*, *j*) and µ is the mean pixel value of the image.
- **Entropy** is the average information of an image that characterizes its texture and is affected by any noise present in the image, as defined in Equation (4). When the entropy is higher, it is expected that the image shows a higher amount of information.^[^ ^68^ ^]^
(4) $$Entropy=-\sum_{i=0}^{n}p\left(a_{i}\right)logp\left(a_{i}\right)$$
  where *p*(*a*_*i*_) shows the histogram of the intensity levels in the image.
- **Spatial Frequency (*f*_*x*_ and *f*_*y*_)** gives information regarding the intensity variation of an image over a distance in space domain (*x* and *y* directon). In other words, spatial frequency refers to the periodicity in which the intensity values of an image changes. Hence, features of an image that show intensity variability in short distance have high spatial frequency. On the contrary, the features that show intensity variability in a large spatial portion of the image have low spatial frequency. Just like time‐domain signals, spatial frequency can be calculated using Fourier transform

### 4.10 Clustering and Material Properties

By calculating the three contrast measures of the resulting images from the MODWPT+CWT technique for the excited frequency, non‐driven frequencies, and the mixing products of all, we obtained a three‐dimensional data set of contrast measures. To better understand the dynamics of the images with consideration of material properties and their non‐linear characteristics, we propose to use data reduction and clustering to identify the images with similar contrast behaviors. To this extent, Ma, R. et al.^[^ ^40^ ^]^ suggested a spectral method to incorporate the results of multiple data visualization and reduction techniques, such as t‐Distributed Stochastic Neighbour Embedding (t‐SNE), Uniform Manifold Approximation and Projection (UMAP), and Principal component analysis (PCA), while maintaining the structural pattern of each data point. This method aims at enhancing the strengths of each candidate visualization while alleviating the weaknesses.

In this study, we used 8 visualizations; i.e., Kernel PCA with two sets of parameters, t‐SNE, and UMAP each with three sets of parameters (more information in Section S4, Supplementary Information). All the 8 visualisations are combined using the methodology, proposed by Ma, R. et al.,^[^ ^40^ ^]^ leading to a new “meta‐visualization” that captures the structures of the data. Having the meta‐visualization, we are able to easily cluster the contrast measures and therefore, describe the material properties considering their associated clusters.

### 4.11 Contrast Enhancement

Owing to the information acquired by the clusters, we can choose the images which describe the material properties and hence reduce the probability of enhancing contrast obtained from artefacts. Further, an image fusion technique is used to combine images of the same cluster and construct a single image while maintaining the original images' main attributes and improving the key features by considering the pixel‐level grouping.

Image fusion is used for multi‐modal imaging where signals for variable imaging techniques are produced simultaneously, or for multi‐sensor imaging where the same image is obtained by multiple sensors or by the same sensor with diverse working modes. Given that multi‐scale and multi‐resolution components would enhance the image fusion, DWT can largely complement the fusion process by increasing texture information. DWT allows for the decomposition of the image into coefficients that maintain the image information (more information in Section S4, Supporting Information). As such, the coefficients obtained from different images can be combined using appropriate fusion rules to form new coefficients containing the information of multiple images. Further, Inverse DWT (IDWT) can be applied to generate the fused image.^[^ ^69^ ^]^

Fusion rules are algorithms that combine two images by identifying the features of interest and detaining the insignificant features in the images. Therefore, the quality of the fused image depends on the appropriate selection of the fusion rule. Principal component analysis (PCA) is a mathematical tool that is used in image fusion.^[^ ^70^ ^]^ In order to use PCA as an image fusion rule, the normalized eigenvector of the covariance matrices for each of the images is calculated. The obtained eigenvalues are further used as weights to calculate the fused image by a weighted sum of the original images.

In this study, we used the DWT‐PCA hybrid image fusion algorithm to combine the images of different harmonics, with the purpose of increasing the image contrast. Based on the DWT‐PCA algorithm, the source images, i.e., different harmonics, are first decomposed into their approximate and detailed components using DWT with “db4” as the mother wavelet to 2 levels of decomposition. Then PCA is applied to the corresponding coefficients of each source image to calculate the normalized eigenvalues. The eigenvalues are further used to calculate the weighted sum of each coefficient as the fusion rule. IDWT is then applied to the calculated fused coefficients to combine the coefficients and reconstruct the image.^[^ ^71^ ^]^

The contrast of the fused images are further evaluated considering the three measures described in Equations (2), (3), and (4).

### 4.12 Histogram‐Based Probability Distribution Function (PDF) Determination

The dataset's probability density function (PDF) for an unknown distribution was obtained using a histogram‐based method in MATLAB. In MATLAB, the data were analyzed using the histogram and *“histcounts”* functions with the *“Normalization*,” and *“pdf”* options to ensure that the area under the histogram equals unity, providing a normalized representation of the data's distribution.

The *“histcounts”* function was utilized to calculate the histogram data, where counts represent the number of data points in each bin, normalized to form a PDF, and edges represent the bin edges.

Mathematically, given a dataset *X* = {*X*_1_, *X*_2_, …, *X*_*N*_} with *N* data points, the bin edges *e* = {*e*_1_, *e*_2_, …, *e*_*M* + 1_} define *M* bins, with the bin width *W*_*i*_ for the *i* ^*th*^ bin calculated as *W*_*i*_ = *e*_*i* + 1_ − *e*_*i*_.

The number of data points *n*_*i*_ falling into each bin *i* is counted, and the PDF f(x) is then normalized as $f_{(}i)=\frac{n_{i}}{\left(N.W_{i}\right)}$ where *f*_*i*_ is the probability density for the *i* ^*th*^ bin.

The as‐obtained histogram data were subsequently fitted using the Gaussian Peak fit function using Origin Software (OriginPro). The Peaks were subsequently integrated to obtain the percentage ratio of each component.

## Conflict of Interest

The authors declare no conflict of interest.

## Supporting information

Supporting Information

## Data Availability

The data that support the findings of this study are available from the corresponding author upon reasonable request.
