# Supplementary material for: Nonlinear Harmonics: A Gateway to Enhanced Image Contrast and Material Discrimination
Source: Adv Sci (Weinh). 2025 Jan 28;12(11):2411556. doi: 10.1002/advs.202411556 (PMC11923995; doi:10.1002/advs.202411556)
Supplement: Supplementary file 1 — Supporting Information [file ADVS-12-2411556-s001.pdf]

ADVANCED SCIENCE
Open Access

Supporting Information

for *Adv. Sci.*, DOI 10.1002/advs.202411556

Nonlinear Harmonics: A Gateway to Enhanced Image Contrast and Material Discrimination

Pardis Biglarbeigi, Gourav Bhattacharya, Dewar Finlay and Amir Farokh Payam**

Supplementary Information - Nonlinear Harmonics: A Gateway to Enhanced Image Contrast and Material Discrimination

Pardis Biglarbeigi[1*], Gourav Bhattacharya[2], Dewar Finlay[2], Amir Farokh Payam[2*]

[1]Department of Pharmacology & Therapeutics, University of Liverpool, Whelan Building, Liverpool, L69 3GE, England, UK.
[2]School of Engineering, Ulster University, York Street, Belfast, BT15 1AP, Northern Ireland, UK.

*Corresponding author(s). E-mail(s): p.biglarbeigi@liverpool.ac.uk; a.farokh-payam@ulster.ac.uk;
Contributing authors: g.bhattacharya@ulster.ac.uk; d.finlay@ulster.ac.uk;

1 Wavelet Transform

Continuous Wavelet Transform (CWT) can be obtained by calculating the convolution of sliding and dilating mother wavelet, $\psi(t)$, and the signal, $x(t)$:

$$W(t,s) = \frac{1}{s}\int \psi^*(\frac{u-t}{s})du \tag{1}$$

In Eq. 1, s is defined as the scale (representative of frequency bands) and t is the time shift of the mother wavelet, and ψ^* represents the complex conjugate of the ψ, where the dilation and translation parameters of ψ varies continuously. CWT uses band-pass filters localised in ω_s to transform the signal into different scales. Generalised Morse Wavelets (GMW) are generally the recommended wavelet family for analysing modulated signals due to their capability of retaining signal characteristics while staying analytical[1]. However, the compressed GMW filters for detecting amplitude of higher harmonics seem to suffer from deficiencies which result in fluctuations and inaccurate amplitude calculation.

Discrete Wavelet Transform (DWT) coefficients can be defined as the sampled CWT coefficients. As such, in DWT the time is continuous, but the scale and time-shift parameters are dyadically discretised into finite numbers. Therefore, DWT breaks the non-stationary signal into finite set of frequency sub-signals. For each level of decomposition, DWT uses mother wavelets interpreted as low and high-pass Finite Impulse Response (FIR) filters along with down-sampling by a factor of 2 to estimate the approximation and detail coefficients, respectively. Although, DWT performs the decomposition only on the approximation coefficients, which makes it unsuitable in further analysing the higher harmonics of a signal. Discrete Wavelet Packet Transform (DWPT), as an extension to DWT, uses wavelet basis functions to create a tree structure of filter banks, which performs the decomposition on both approximation and detail coefficients and accordingly results in homogeneous frequency bands. Yet, the down-sampling process in both DWT and DWPT makes them time-variant transforms. By avoiding the down sampling process, and use of filter banks for both detail and approximation coefficients, MODWPT can be a suitable mean in estimating the time-invariant sub-signal of higher harmonics [2].
Mathematically, MODWPT coefficients, $W_{(j,n,t)}$ at level j, time t, and frequency band n (where $n = 0, 1, \dots, 2^{j-1}$) is obtained by convolution of the signal $x = W_{(0,0,t)}$ and the mother wavelet interpreted as Infinite Impulse Response (IIR) high and low-pass filters are defined in Eqs 2, and 3 :

$$W_{i,n,t} = \sum_{l=0}^{L-1} \tilde{f}_{n,l} W_{j-1,\frac{n}{2},(t-2^{j-1}l)} \mod N \qquad t = 0, 1, ..., N-1 \tag{2}$$

$$\tilde{f}_{n,l} = \begin{cases} g_l/\sqrt{2}, & if \quad n \mod 4 = \quad or \quad 3 \\ h_l/\sqrt{2}, & if \quad n \mod 4 = 1 \quad or \quad 4 \end{cases} \tag{3}$$

where L is the length of the wavelet filter (i.e., mother wavelet), and mod is the modulo operator. Furthermore, g_l, and h_l are quadrature mirror filters defined as Eq. 4.

$$\begin{aligned} h_l &= (-1)^l g_{L-l-1} \qquad l = 0, 1, ..., L-1 \\ g_l &= (-1)^{l+1} h_{L-l-1} \qquad l = 0, 1, ..., L-1 \end{aligned} \tag{4}$$

The choice of mother wavelet in MODWPT is an important factor. Mother wavelets are characterised by their symmetry, orthogonality, compactness, and decay. However, Guido, R.C [3] has suggested that if the application of the WT does not require implementation of the inverse of WT, the choice of mother wavelet solely depends on frequency and phase response of the associated filter banks. For calculation of higher harmonic amplitudes, the MODWPT is only used to decompose the signal into time-invariant sub-signals with a specific frequency band. Therefore, high order Daubechies wavelet packets are suitable choice for mother wavelet as they provide smoother magnitude response [4]. Accordingly, in this study, 'db4' is used as the mother wavelet to provide smooth decomposition. After the decomposition of the photodetector signal into frequency-band sub-signals, CWT can be used to obtain the amplitude of the harmonics associated with each of the frequency bands.

Moreover in recent years, DWT has increasingly been used in image processing. Images are considered to be two-dimensional signals where DWT is applied to filter the rows and columns of the image. As the application of low- and high-pass filtering on the images contains redundant processes, there is a need for down-sampling step after filtering. This results in the decomposition of the complex information of the image into four components with different position and scales; i.e., the lower resolution approximation image (LL) which shows the trend of pixel values, along with the detail components in horizontal (HL), vertical (LH), and diagonal (HH) components in which the edges and lines of the image in horizontal, vertical, and diagonal are more notable, respectively. These components can further be used in reconstructing the image with higher precision [5,6].

2 Mixing harmonic products

Following the extraction of time-frequency information of the time-domain signals, using the methodology developed in previous work [7], the resulting amplitude of the signal at the cantilever excitation frequency f_1, eigen frequency f_2, the second to sixth harmonics as well as a set of mixing products of all, are used to reconstruct the images. A total of $n = 30$ different of the harmonics and mixing product images are considered as listed in Table S1.

Table S1 The considered images of the harmonics and mixing product obtained from WT-AFM methodology [7]

f_1	$f_1 - 6f_1$	$6f_1 - f_2$
$2f_1$	$f_1 + f_2$	$D = f_2 - 6f_1$
$5f_1$	$f_1 - f_2$	$f_1 - D$
$6f_1$	$2f_1 + 6f_1$	$f_1 + D$
f_2	$2f_1 - 6f_1$	$2f_1 - D$
$f_1 - 2f_1$	$2f_1 + f_2$	$2f_1 + D$
$f_1 + 2f_1$	$2f_1 - f_2$	$f_1 - 2D$
$f_1 - 5f_1$	$6f_1 + 5f_1$	$f_1 + 2D$
$f_1 + 5f_1$	$6f_1 - 5f_1$	$cos(\phi)$
$f_1 + 6f_1$	$6f_1 + f_2$	$sin(\phi)$

3 Results obtained from WT-AFM prior to any normalisation and post-processing

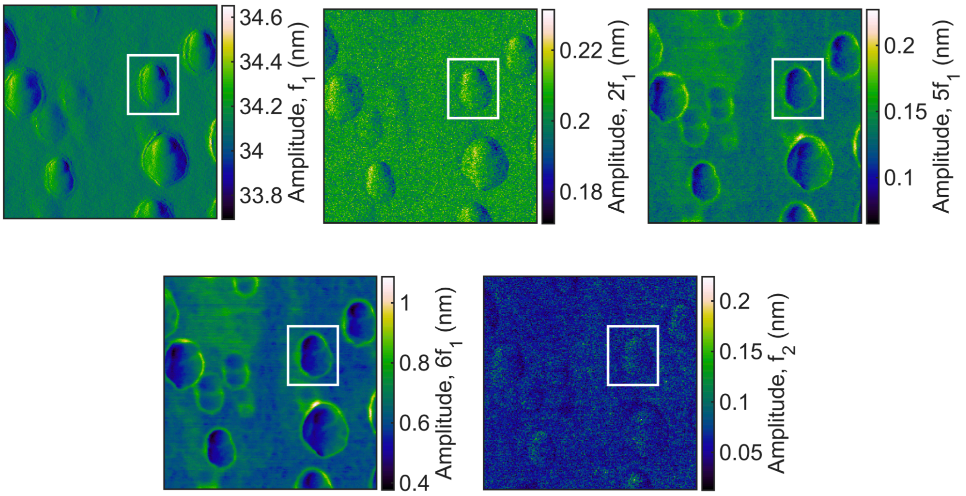

Fig. S1 SWCNT sample. An area of $0.34 \times 0.34 \mu m$ of the sample is investigated. Each figure represents harmonic amplitude of the sample obtained from WT-AFM method.

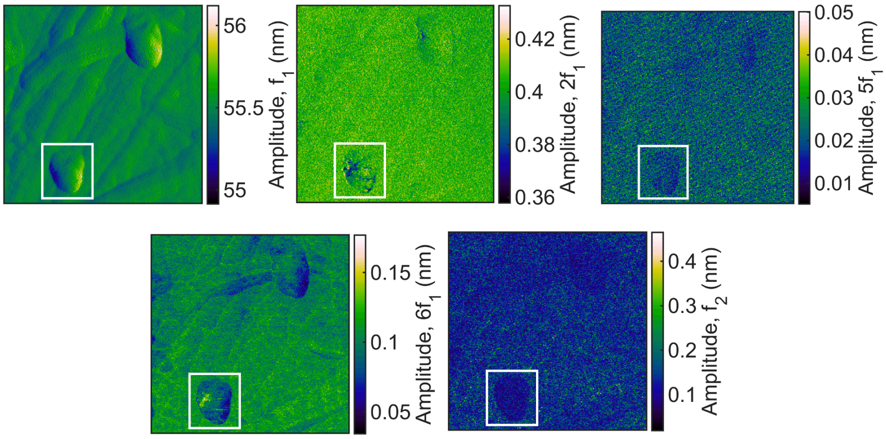

Fig. S2 GO-HOPG sample. An area of $0.78 \times 0.78 \mu m$ of the sample is investigated. Each figure represents harmonic amplitude of the sample obtained from WT-AFM method.

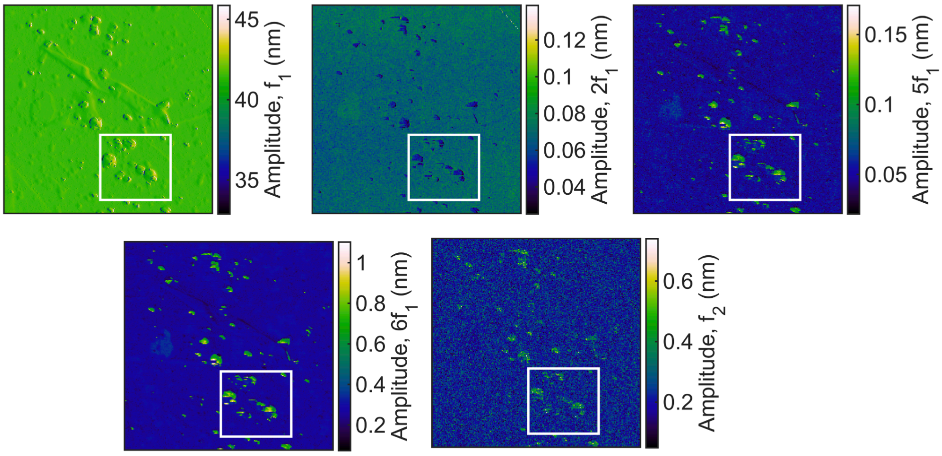

Fig. S3 Deposited Au- nanoparticles on multi-layer HOPG sample. An area of $2 \times 2 \mu m$ of the sample is investigated. Each figure represents harmonic amplitude of the sample obtained from WT-AFM method.

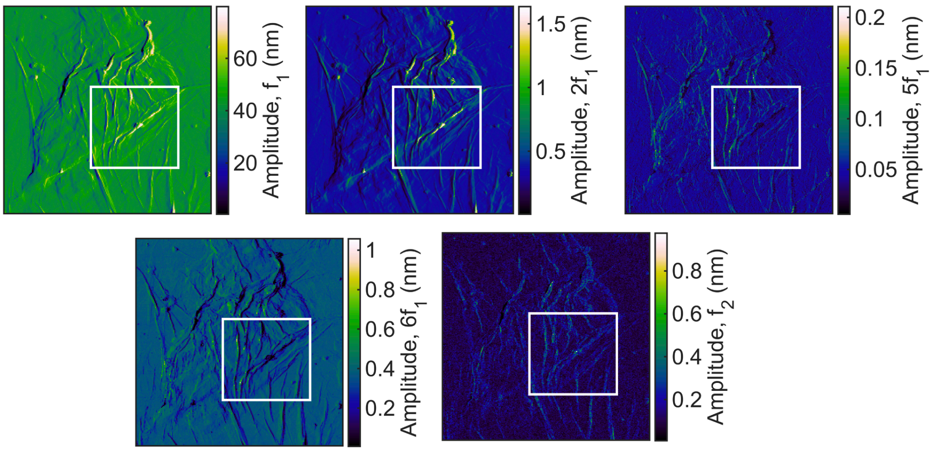

Fig. S4 Deposited Au- nanoparticles on multi-layer GO-HOPG sample. An area of $12 \times 12 \mu m$ of the sample is investigated. Each figure represents harmonic amplitude of the sample obtained from WT-AFM method.

4 Data Visualisation

- **Kernel Principal Component Analysis (kPCA)** is extension of the widely-used feature extraction algorithm of PCA. The limitation in the application of PCA lies in its linear data separation nature. This limitation can be overcome by use of kPCA which makes it possible to be used for non-linear dimensionality reduction [8]. In kPCA, if we assume the non-linear mapping of the n variables called x is represented by $\phi(x)$, it is shown that the kernel function of $\phi(x)$, $\kappa(x) = \phi(x)\phi^T(x)$, plays the role of covariance matrix in linear PCA. Further, the eigen values and principal components can be further calculated from $\kappa(x)$. There are different choices for kernel matrix. In this work, we used two sets of Gaussian and Laplacian kernels [9] to reduce the dimensionality of contrast measures matrices to aid in data exploratory analysis.

- **t-Distributed Stochastic Neighbor Embedding (t-SNE)** is a variation of SNE that converts a high-dimensional dataset into 2 or 3 dimensions. SNE converts the high-dimensional Euclidean distance between the data points of x into a representation of similarity. The similarity of two datapoints, x_i and x_j, can be described as the conditional probability of x_i and x_j being in the same neighbourhood, where the neighbourhood is considered as a Gaussian probability density function centered at x_i. The performance of the similarity between x_i and x_j is calculated by a cost function. Therefore, the aim of SNE is to minimise the cost function to obtain a suitable dimensionality reduction. However, the optimisation of the cost function associated with SNE is problematic. Thus, to mitigate this problem, Van der Maaten, L. and Hinton, G. [10] suggested t-SNE that uses symmetric cost function and Student-t distribution rather than Gaussian for similarity calculation in the lower-dimensional dataset. t-SNE, further allows customisation of the calculation of distance between datapoints. In this study, we used MATLAB in-built function with three sets of t-SNE visualisations considering Euclidean distance between datapoints, cosine of the angle between the datapoints, and the linear correlation between the datapoints.

- **Uniform Manifold Approximation and Projection (UMAP)** is introduced based on manifold theory and topological data analysis, and is acclaimed to preserve the global structure of the dataset [11]. UMAP's operation is based on weighted graphs and k-neighbour based graph learning algorithms. UMAP, like t-SNE, allows customisation of distance calculations. Thus, in this study, we used three visualisations obtained from UMAP algorithm [12] adopting Euclidean distance, cosine of the angle, and the linear correlation between the datapoints.

5 Field Emission Scanning Electron Microscopy (FESEM)-Energy-Dispersive X-ray Spectroscopy (EDX) Results

To verify the accuracy of our methodology and confirm that the detected features in our imaging are indeed the ones we claim, we have performed a detailed SEM-EDX analysis on all the samples used in our AFM measurement.

In the first sample, SWCNT dispersion (dispersed in IPA) was deposited onto a silicon substrate featuring a native oxide layer. The FE-SEM image illustrates the distribution of carbon nanotubes on the substrate, while EDX mapping confirms the elemental composition. Specifically, the $C_{k\alpha}$ spectra validate the presence of carbon attributable to SWCNTs, whereas the $Si_{k\alpha}$ and $O_{k\alpha}$ spectra correspond to the silicon substrate, corroborating the reliability of AFM measurements. Additionally, the EDS spectrum reveals a carbon content of approximately 4%, providing further confirmation of the presence of SWCNTs in the sample.

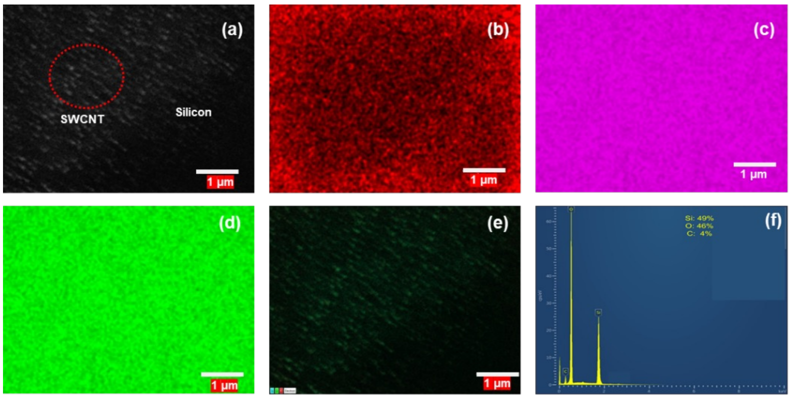

Fig. S5 (a) FE-SEM image of SWCNT deposited over Silicon substrate with native oxide layer, corresponding EDX map of (b) $C_{k\alpha}$ spectra, (c) $O_{k\alpha}$ spectra (d) $Si_{k\alpha}$ spectra,(e) the composite and (f) the corresponding elemental percentage

In the HOPG sample, the SEM image clearly shows the layered structure with visible terraces (Fig. S6), characteristic of HOPG. Since HOPG is composed solely of carbon, only carbon ($C_{k\alpha}$) is detected in the EDX spectra, confirming the composition.

Next, we consider the HOPG-GO sample. The SEM-EDX spectra is represented in Fig. S7. In the HOPG-GO sample, the FE-SEM image reveals distinct microstructural features of both the HOPG backbone and the GO regions (highlighted by the yellow dotted area). The EDX map of $C_{k\alpha}$ confirms the presence of carbon across both HOPG and GO regions.

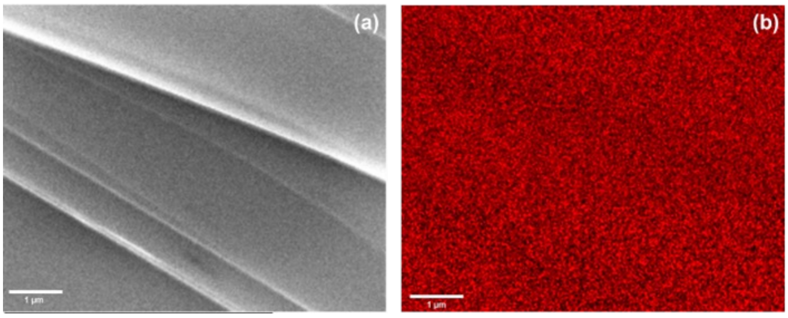

Fig. S6 (a) FE-SEM image of HOPG (b) the corresponding EDX map of $C_{k\alpha}$ spectra.

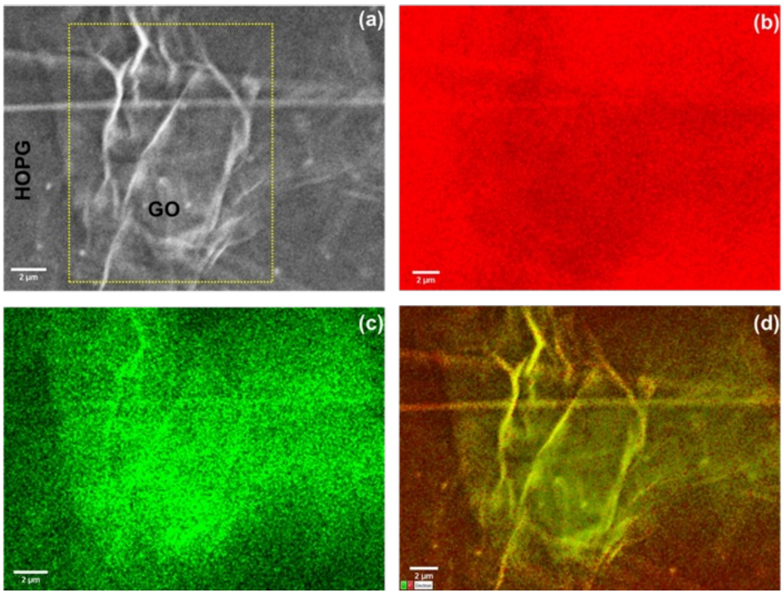

Fig. S7 (a) FE-SEM image of HOPG- GO, corresponding EDX map of (b) $C_{k\alpha}$ spectra, (c) $O_{k\alpha}$ spectra, (d) the composite.

Additionally, the $O_{k\alpha}$ map shows increased oxygen content in the GO regions, particularly near edge planes, consistent with the oxygen-containing functional groups in GO. This result confirms that the sample under investigation in our AFM study is indeed GO-decorated HOPG.
In the next step, we considered Au NP decorated HOPG sample and the SEM micrograph along with the EDX elemental mapping and represented in Fig. S8.

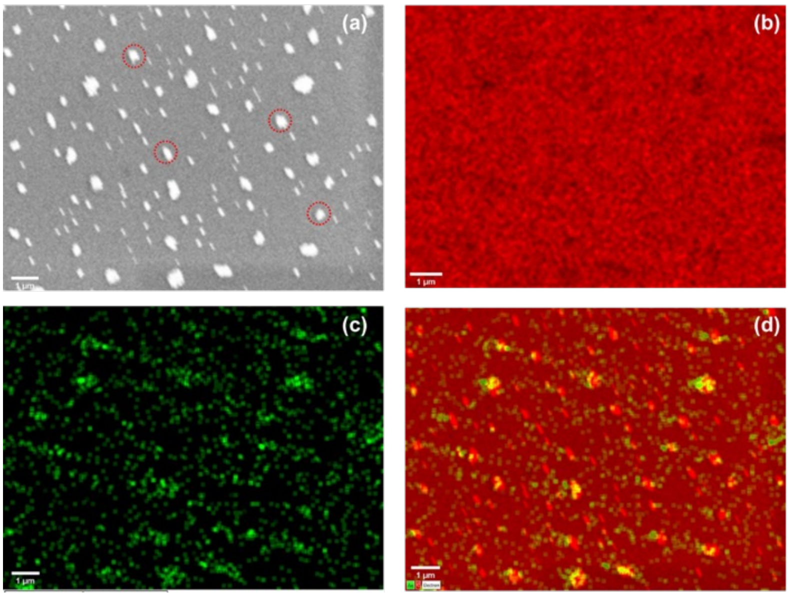

Fig. S8 FE-SEM image of HOPG- Au, corresponding EDX map of (b) $C_{k\alpha}$ spectra, (c) $Au_{M\alpha}$ spectra, (d) the composite.

In the Au NP-decorated HOPG sample, the FE-SEM micrograph clearly shows Au nanoparticles (NPs) on the HOPG substrate. EDX mapping confirms the presence of carbon ($C_{k\alpha}$) from the HOPG substrate, while the $Au_{M\alpha}$ spectra confirm the distribution of Au NPs across the substrate. This further supports the presence of Au NPs in our imaging.
In the last step we obtained the FESEM image of the Au NP and GO decorated HOPG sample and the corresponding EDX elemental mapping and represented in Fig. S9.

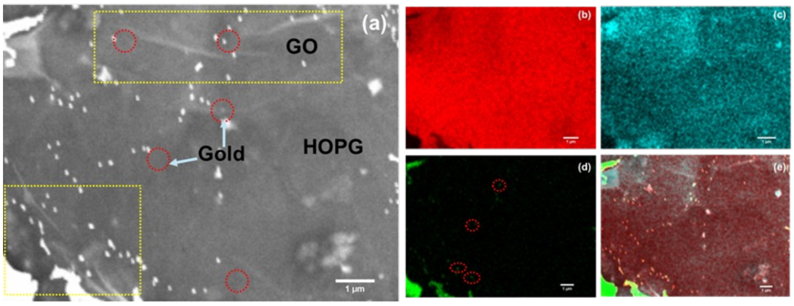

Fig. S9 FE-SEM image of GO-HOPG- Au, corresponding EDX map of (b) $C_{k\alpha}$ spectra, (c) $O_{k\alpha}$ spectra (d) $Au_{M\alpha}$ spectra, and (e) the composite.

In the final sample, where both Au NPs and GO flakes decorate the HOPG substrate, the FE-SEM image shows the distribution of both components on the HOPG. EDX

maps verify the elemental composition: $C_{k\alpha}$ spectra confirm carbon in both HOPG and GO regions, $O_{k\alpha}$ spectra reveal increased oxygen content at GO-decorated sites, and $Au_{M\alpha}$ spectra indicate the presence of Au NPs at specific sites. These findings confirm that the sample comprises all three constituents (HOPG, GO, and Au NPs), validating the accuracy of our AFM measurements.
In summary, this comprehensive SEM-EDX analysis substantiates the presence of Au NPs and GO flakes on the HOPG substrate and confirms the correctness of our AFM measurements and the enhancements achieved through our image fusion method.

6 Effective clusters through AFM-ICE

After applying the proposed AFM-ICE second step, "the effective cluster", which presents the images showing better contrast according to numerical considerations and the calculated image contrast measures, for each sample is defined. Tables S2, S3, S4, and S5 represent the resulting effective clusters for GO-HOPG, deposited nanoparticles on multi-layer HOPG, and deposited nanoparticles on multi-layer GO-HOPG samples, respectively.

Table S2 Effective cluster for SWCNT sample

$2f_1$	$6f_1 + 5f_1$
$5f_1$	$6f_1 - 5f_1$
$6f_1$	$6f_1 + f_2$
f_2	$6f_1 - f_2$
$f_1 - 5f_1$	D
$f_1 - 6f_1$	$f_1 + D$
$2f_1 + 6f_1$	$2f_1 - D$
$2f_1 - 6f_1$	$2f_1 + D$
$2f_1 + f_2$	$f_1 - 2D$
$2f_1 - f_2$	$cos(\phi)$
$sin(\phi)$	

Table S3 Effective cluster for GO-HOPG sample

$2f_1$	$6f_1 + 5f_1$
$5f_1$	$6f_1 - 5f_1$
$6f_1$	$6f_1 + f_2$
f_2	$6f_1 - f_2$
$f_1 + 6f_1$	D
$f_1 + f_2$	$f_1 + D$
$2f_1 + 6f_1$	$2f_1 - D$
$2f_1 - 6f_1$	$2f_1 + D$
$2f_1 + f_2$	$f_1 - 2D$
$2f_1 - f_2$	$f_1 + 2D$

Table S4 Effective cluster for deposited Au-nanoparticles on multi-layer HOPG sample

$2f_1$	$6f_1 + f_2$
$5f_1$	$6f_1 - f_2$
f_2	D
$2f_1 + f_2$	$2f_1 - D$
$2f_1 - f_2$	$2f_1 + D$

Table S5 Effective cluster for deposited Au-nanoparticles on multi-layer GO-HOPG sample

$5f_1$	$6f_1 + f_2$
$6f_1$	$6f_1 - f_2$
f_2	D
$2f_1 - 6f_1$	$2f_1 - D$
$2f_1 + f_2$	$2f_1 + D$
$6f_1 + 5f_1$	$sin(\phi)$
$6f_1 - 5f_1$	

7 Structural Analysis and Validity of AFM-ICE

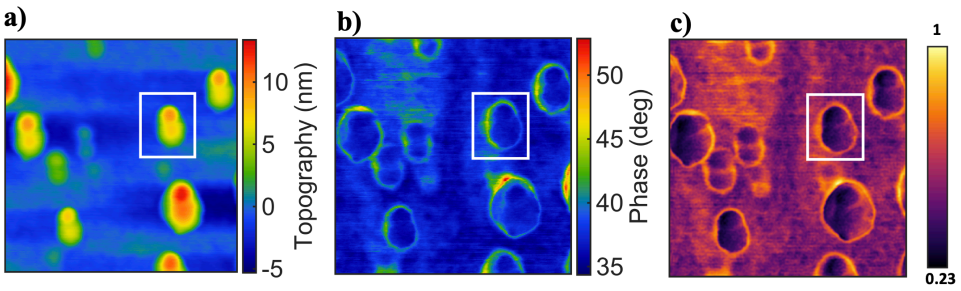

Fig. S10 SWCNT sample (a) Topography , (b) Phase obtained from LIA, and (c) resulted AFM-ICE image.

Table S6 Linear correlation between harmonic, mixing product of harmonics, the AFM-ICE image, and the topography obtained from LIA for the ROI represented in Fig S10.

	Topography (ROI)		Topography (ROI)
f_1	-0.17	f_2	0.26
$2f_1$	-0.12	$6f_1 - 5f_1$	-0.54
$5f_1$	-0.42	$6f_1 - f_2$	-0.55
$6f_1$	-0.55	**AFM-ICE**	**-0.58**

8 Histograms and contrast measure (c) fits

Fig. S11 illustrates the histogram of WT-AFM reconstructed images for f_1, $2f_1$, $5f_1$, $6f_1$, and f_2 for SWCNT. As can be seen none of the fitted histograms show the contrast. However, a small second peak can be seen in the image obtained from AFM-ICE, illustrating the improvement in the contrast and material discrimination.

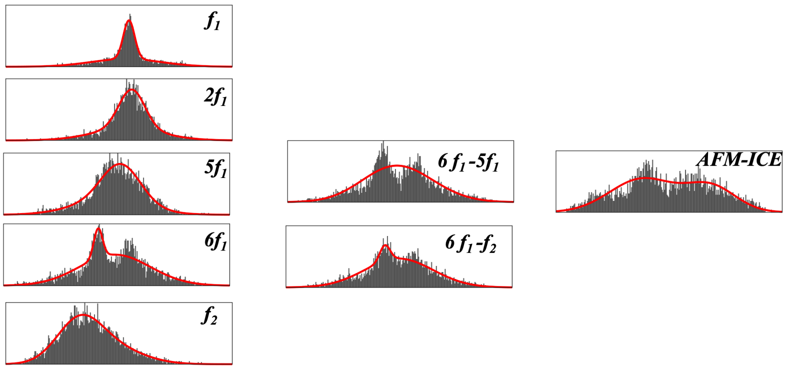

Fig. S11 SWCNT sample. WT-AFM reconstructed image histogram and the fitted GMM for the ROI (i.e. summation of 2 normal distributions).

Fig. S12 illustrates the histogram of WT-AFM reconstructed images for $f_1, 2f_1, 5f_1, 6f_1$, and f_2. As can be seen $2f_1$ and $6f_1$ images show some contrast for the division of the materials in the ROI. AFM-ICE histogram a very clear distinction between the two materials as visualised in its histogram.

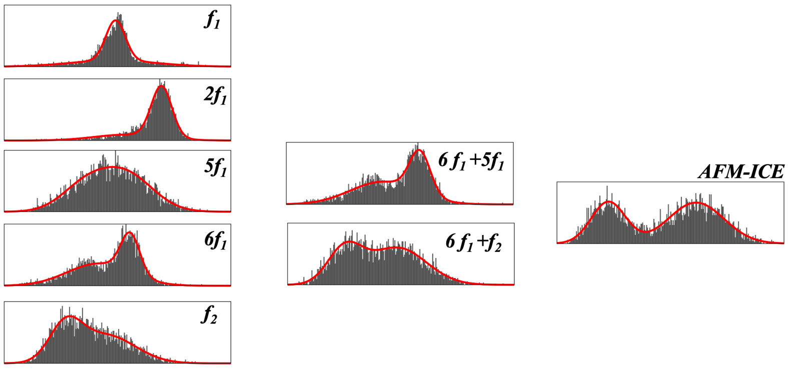

Fig. S12 GO-HOPG sample. WT-AFM reconstructed image histogram and the fitted GMM for the ROI (i.e. summation of 2 normal distributions).

Fig. S13 represents the histogram of WT-AFM reconstructed images for $f_1, 2f_1, 5f_1, 6f_1$, and f_2. As can be seen, none of the harmonics, or mixture of them

show any valuable information for the nanoparticles of Gold deposited on the sample. However, AFM-ICE was successful in extracting some information in the image, as visualised in its histogram and shown with an arrow. It is clear that since the size of the particles is much lower than HOPG in ROI and in the overall sample, the count for pixels containing the Gold nanoparticles are very small compared to the size of the image.

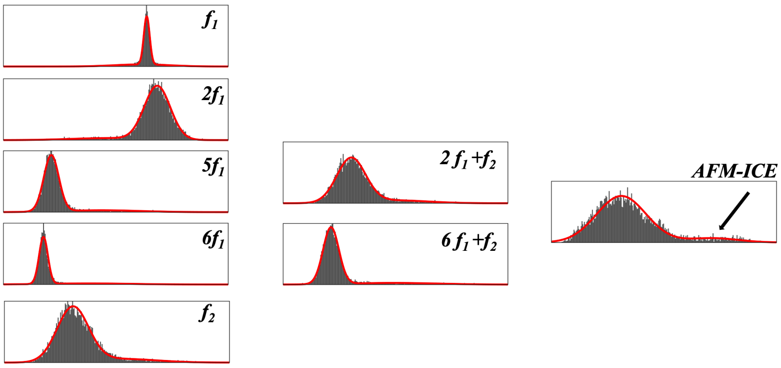

Fig. S13 Deposited Au-nanoparticles on multi-layer HOPG sample. WT-AFM reconstructed image histogram and the fitted GMM for the ROI (i.e. summation of 2 normal distributions).

Fig. S14 represents the histogram of WT-AFM reconstructed images for $f_1, 2f_1, 5f_1, 6f_1$, and f_2. It is obvious from the images, that none of them are able to show the details of the materials in the sample, as can be seen in their histograms of ROI. $6f_1$, and f_2 represent the presence of 2 materials, however, not with very clear contrast. As the result of the fusion of the two chosen images from effective cluster, three peaks can clearly be seen in the histogram of the AFM-ICE reconstructed images. Therefore, fitting a GMM with considering 2 normal distributions is not able to represent the image histogram with acceptable accuracy (shown as RED fitted histogram in the AFM-ICE reconstructed image). We considered fitting a GMM with 3 normal distributions (shown in GREEN fitted histogram in the AFM-ICE reconstructed image). As can be seen from the histogram, not only numerically this is a better fit for the histogram, but also it confirms the presence of three materials (i.e. GO, HOPG, Gold nanoparticles) in the sample. This implies that the proposed AFM-ICE methodology can be used as an effective post-hoc analysis to enhance AFM images. Our method combines the material property considerations (i.e. harmonics) with numerical methods to increase the contrast of the AFM images while extracting more information from the images that may not be achieved from the conventional LIA images.

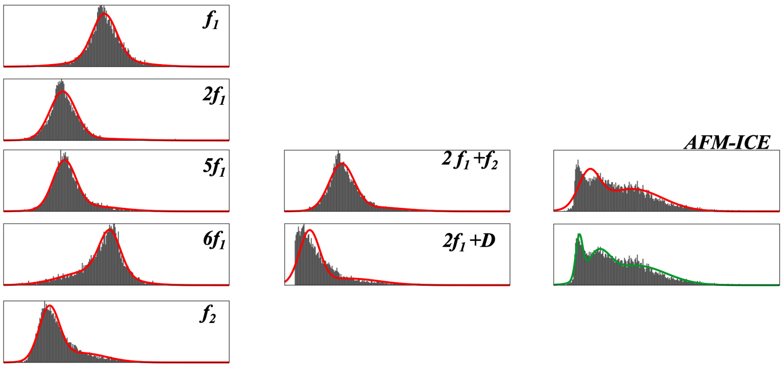

Fig. S14 Deposited Au-nanoparticles on multi-layer GO-HOPG sample. WT-AFM reconstructed image histogram and the fitted GMM for the ROI (i.e. summation of 2 normal distributions, represented in RED and summation of 3 normal distributions, represented in GREEN).

9 Multiple scan areas and statistical test

To test the robustness of the methodology, we scanned different areas on the samples and applied the AFM-ICE framework to enhance the contrast of AFM images. We further considered 4 different regions of interest (ROIs) with variable surface areas, on each of the samples for detailed analysis. The three contrast measures of c (obtained from image histograms), standard deviation, and entropy were calculated for each of the ROIs of the two selected images from effective cluster before fusion, and for the resulted image after fusion. We further applied a one-way analysis of variance (ANOVA) test on the contrast measures. ANOVA is a statistical test that is used to analyse the difference between the means of more than two samples. In one way ANOVA, one of the samples are considered to be independent. The p-value is calculated in two occasions, one with considering only the two images selected from effective cluster as the entries, and the other considering the two original images as well as the fused image obtained from AFM-ICE. For these two tests, the null hypothesis claims that the image entries do not affect the contrast measures of the image in different ROIs. Figures S15, S16, S17, and S18 represent the results obtained from further analysis of variable ROIs and scan areas for the three samples under study. The results show that the three contrast measures are constantly higher for images obtained from AFM-ICE, illustrating better contrast in these images. Further, the p-values obtained from the original images selected from the effective clusters show higher values (i.e., $p - value > 0.05$ for at least 1 measure in each sample). These results fail to reject the null hypothesis, indicating that the images do not affect the contrast measures in the ROIs. However, when considering the images obtained from AFM-ICE, p-value is constantly lower than p-value associated with original images from effective cluster (in most cases less than 0.05 and even ~ 0 in some cases), resulting in the rejection of the null hypothesis, and showing statistically significant improvement in the contrast of the images in different regions.

It is important to note that to show the robustness of our proposed AFM-ICE framework, we took the same images (i.e., same mixing harmonics) from the effective cluster for each sample as entries for image fusion step, even for different scanned areas of the sample. Our proposed methodology demonstrated a statistically significant improvement in the contrast enhancement of the images. However, given that the proposed methodology is data-driven and clearly the histogram of the images vary with changing the scanning area, the same image entries of the effective cluster may not show the best results in different scanning areas. Therefore, it is important to choose two images from the effective cluster that would result in the best fused images. The selection of the images from effective cluster, can be easily done by observation. As an example, Figure S17.b. shows very limited information in the mixing harmonic of $2f_1 + f_2$. This might be mainly due to the larger size of the scanned area and the proportionally small gold particles in the image. Therefore, more careful selection of image entries from the effective cluster may result in best results and the highest contrasts.

a)

I.

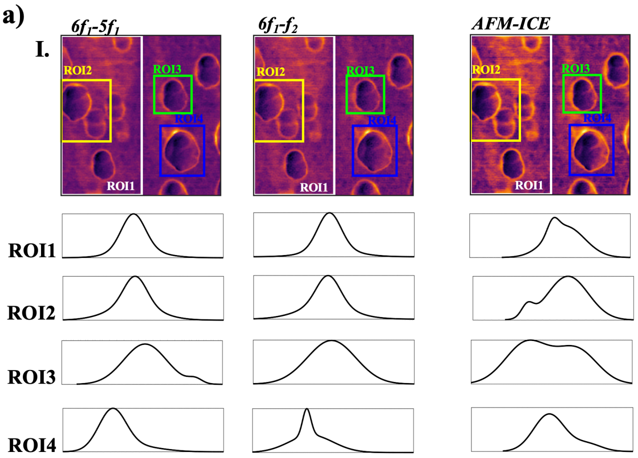

II. p-value (ANOVA)

	Original Images	+ AFM-ICE
c	0.680	0.019
STD	0.893	0.021
Entropy	0.883	0.026

III.

		$6f_1$-$5f_1$	$6f_1$-f_2	*AFM-ICE*
ROI1	c	2.01	1.17	**5.26**
	STD	0.11	0.11	**0.16**
	Entropy	6.79	6.80	**7.36**
ROI2	c	0.91	1.01	**15.36**
	STD	0.13	0.14	**0.19**
	Entropy	7.06	7.11	**7.59**
ROI3	c	4.97	8.52	**16.24**
	STD	0.17	0.17	**0.22**
	Entropy	7.41	7.37	**7.74**
ROI4	c	9.19	1.90	**14.35**
	STD	0.13	0.13	**0.18**
	Entropy	6.91	7.00	**7.48**

b)

I.

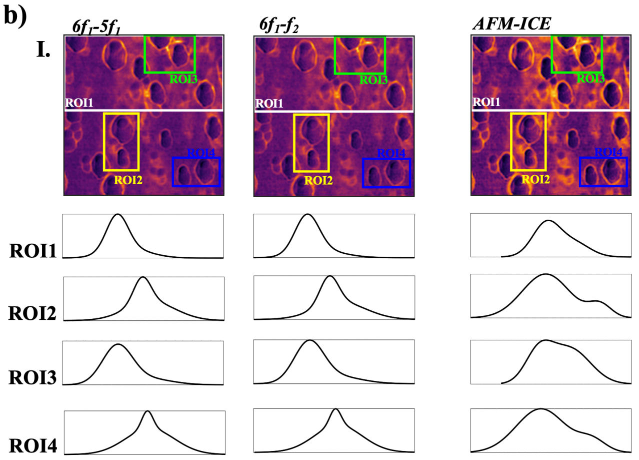

II. p-value (ANOVA)

	Original Images	+ AFM-ICE
c	0.787	0.006
STD	1.000	0.012
Entropy	0.962	0.038

III.

		$6f_1$-$5f_1$	$6f_1$-f_2	*AFM-ICE*
ROI1	c	6.38	4.41	**14.49**
	STD	0.11	0.11	**0.16**
	Entropy	6.80	6.77	**7.35**
ROI2	c	4.25	2.96	**19.84**
	STD	0.15	0.15	**0.20**
	Entropy	7.18	7.17	**7.58**
ROI3	c	9.95	10.42	**10.54**
	STD	0.14	0.14	**0.20**
	Entropy	7.10	7.11	**7.54**
ROI4	c	1.28	0.98	**17.24**
	STD	0.16	0.16	**0.20**
	Entropy	7.28	7.28	**7.32**

Fig. S15 SWCNT sample. a) An area of $0.34 \times 0.34\mu m$ and **b)** an area of $0.5 \times 0.5\mu$ of the sample are investigated. I. represents the two selected images from the effective cluster, the resulted image from AFM-ICE image fusion step, the ROIs considered for further analysis, and their associated histograms. II. contains the p-values obtained from one-way ANOVA test, and III. represents the calculated contrast measures for each ROI.

References

[1] Van Drongelen, W.: Signal processing for neuroscientists. Academic press (2018)

[2] Shrifan, N.H.M.M., Akbar, M.F, Isa, N.A.M: Maximal overlap discrete wavelet-packet transform aided microwave nondestructive testing. NDT & E International **119**, 102414 (2021)

[3] Guido, R.C: A note on a practical relationship between filter coefficients and scaling and wavelet functions of discrete wavelet transforms. Applied Mathematics

a)

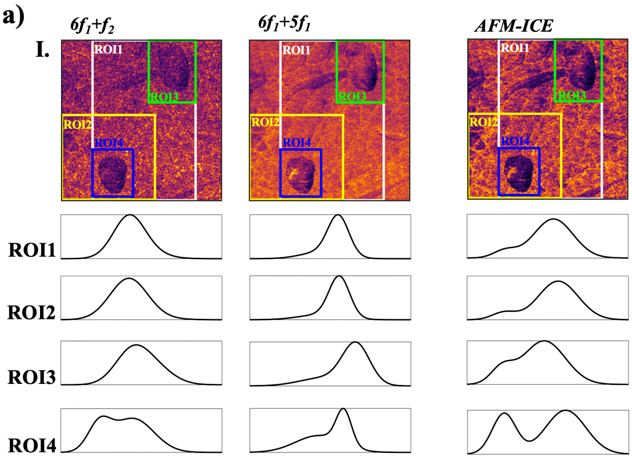

II. p-value (ANOVA)

	Original Images	+ AFM-ICE
c	0.62	0.001
STD	0.58	0.009
Entropy	0.18	0.037

III.

		$6f_1+f_2$	$6f_1+5f_1$	*AFM-ICE*
ROI1	c	6.79	9.87	**23.15**
	STD	0.12	0.10	**0.16**
	Entropy	6.96	6.67	**7.39**
ROI2	c	13.16	9.67	**25.10**
	STD	0.13	0.11	**0.17**
	Entropy	7.12	6.65	**7.45**
ROI3	c	10.91	12.78	**17.89**
	STD	0.14	0.15	**0.17**
	Entropy	7.19	7.17	**7.45**
ROI4	c	17.36	11.07	**28.22**
	STD	0.16	0.15	**0.24**
	Entropy	7.35	7.13	**7.69**

b)

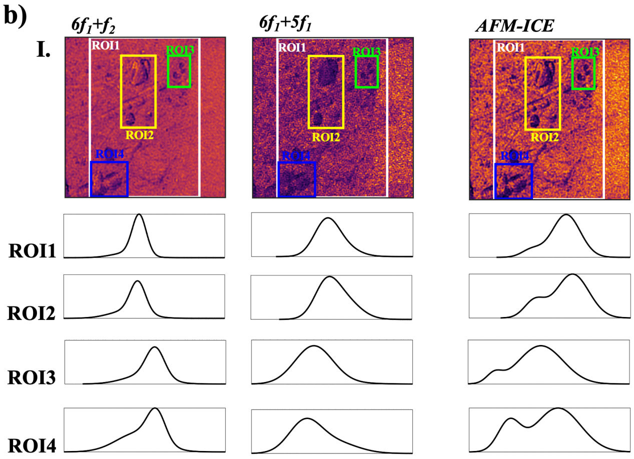

II. p-value (ANOVA)

	Original Images	+ AFM-ICE
c	0.962	~0
STD	0.093	0.013
Entropy	0.053	0.005

III.

		$6f_1+f_2$	$6f_1+5f_1$	*AFM-ICE*
ROI1	c	7.87	10.78	**24.37**
	STD	0.12	0.07	**0.14**
	Entropy	6.96	6.11	**7.21**
ROI2	c	8.02	6.31	**20.60**
	STD	0.14	0.09	**0.18**
	Entropy	7.12	6.47	**7.51**
ROI3	c	6.91	6.60	**19.58**
	STD	0.14	0.12	**0.17**
	Entropy	7.06	6.82	**7.36**
ROI4	c	10.51	9.89	**18.55**
	STD	0.16	0.14	**0.20**
	Entropy	7.29	7.01	**7.59**

Fig. S16 GO-HOPG sample. a) An area of $0.78 \times 0.78\mu m$ and **b)** an area of $2.4 \times 2.4\mu$ of the sample are investigated. I. represents the two selected images from the effective cluster, the resulted image from AFM-ICE image fusion step, the ROIs considered for further analysis, and their associated histograms. II. contains the p-values obtained from one-way ANOVA test, and III. represents the calculated contrast measures for each ROI.

Letters **24**(7), 1257–1259 (2011)

[4] Pham, V.L., Wong, K.P.: Wavelet-transform-based algorithm for harmonic analysis of power system waveforms. IEE Proceedings-Generation, Transmission and Distribution **146**(3), 249–254 (1999)

[5] Nair, R.R., Singh, T.: Multi-sensor medical image fusion using pyramid-based dwt: a multi-resolution approach. IET Image Processing **13**(9), 1447–1459 (2019)

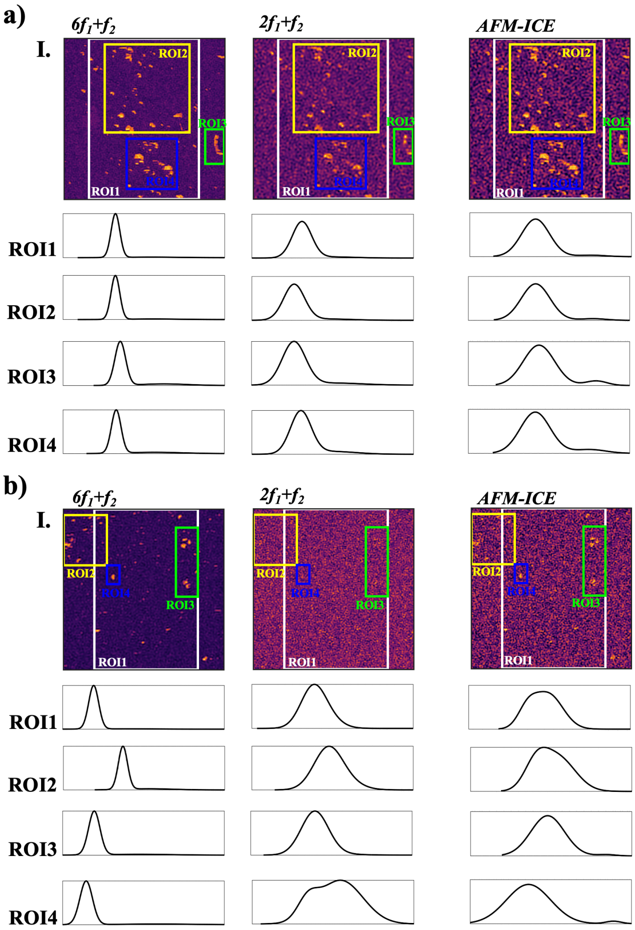

a) II. p-value (ANOVA)

	Original Images	+ AFM-ICE
c	0.046	~ 0
STD	0.601	0.023
Entropy	0.010	~ 0

a) III.

		$6f_1+f_2$	$2f_1+f_2$	*AFM-ICE*
ROI1	c	7.86	11.52	**31.19**
	STD	0.09	0.09	**0.14**
	Entropy	5.53	6.38	**7.05**
ROI2	c	8.58	12.84	**33.64**
	STD	0.09	0.10	**0.15**
	Entropy	5.55	6.52	**7.11**
ROI3	c	11.90	11.28	**32.89**
	STD	0.15	0.13	**0.18**
	Entropy	6.20	6.70	**7.27**
ROI4	c	7.67	11.06	**24.31**
	STD	0.14	0.11	**0.18**
	Entropy	6.11	6.63	**7.31**

b) II. p-value (ANOVA)

	Original Images	+ AFM-ICE
c	0.233	0.114
STD	0.498	0.117
Entropy	0.001	~ 0

b) III.

		$6f_1+f_2$	$2f_1+f_2$	*AFM-ICE*
ROI1	c	7.69	10.22	**13.96**
	STD	0.06	0.10	**0.14**
	Entropy	5.40	6.69	**7.12**
ROI2	c	7.12	9.29	**10.00**
	STD	0.11	0.13	**0.16**
	Entropy	5.94	7.03	**7.31**
ROI3	c	7.47	10.26	**31.27**
	STD	0.12	0.11	**0.16**
	Entropy	5.91	6.77	**7.25**
ROI4	c	11.64	25.24	**34.92**
	STD	0.15	0.17	**0.17**
	Entropy	5.75	7.22	**7.10**

Fig. S17 Deposited Au-nanoparticles on multi-layer HOPG sample. a) An area of $2 \times 2\mu m$ and **b)** an area of $2.74 \times 2.74\mu$ of the sample are investigated. I. represents the two selected images from the effective cluster, the resulted image from AFM-ICE image fusion step, the ROIs considered for further analysis, and their associated histograms. II. contains the p-values obtained from one-way ANOVA test, and III. represents the calculated contrast measures for each ROI.

[6] Pimpalkhute, V.A., Page, R., Kothari, A., Bhurchandi, K.M, Kamble, V.M.: Digital image noise estimation using dwt coefficients. IEEE transactions on image processing **30**, 1962–1972 (2021)

[7] Payam, A.F., Biglarbeigi, P., Morelli, A., Lemoine, P., McLaughlin, J., Finlay, D.: Data acquisition and imaging using wavelet transform: a new path for high speed transient force microscopy. Nanoscale Advances **3**(2), 383–398 (2021)

[8] Schölkopf, B., Smola, A., Müller, K.R.: Kernel principal component analysis.

a)

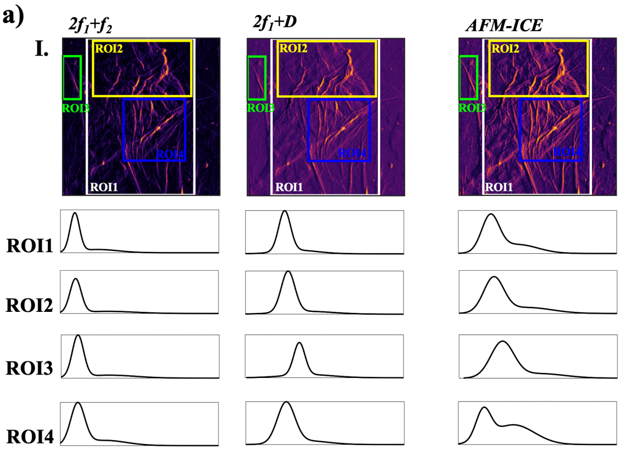

II. p-value (ANOVA)

	Original Images	+ AFM-ICE
c	0.032	0.003
STD	0.031	0.002
Entropy	0.325	~ 0

III.

		$2f_1$+f_2	$2f_1$+D	AFM-ICE
ROI1	c	8.04	4.61	**9.05**
	STD	0.11	0.09	**0.13**
	Entropy	5.98	6.13	**6.84**
ROI2	c	6.07	3.72	**7.40**
	STD	0.14	0.11	**0.16**
	Entropy	6.18	6.36	**6.97**
ROI3	c	6.37	2.06	**8.42**
	STD	0.14	0.11	**0.16**
	Entropy	6.21	6.30	**6.99**
ROI4	c	7.97	6.43	**10.02**
	STD	0.12	0.10	**0.16**
	Entropy	6.41	6.48	**7.13**

b)

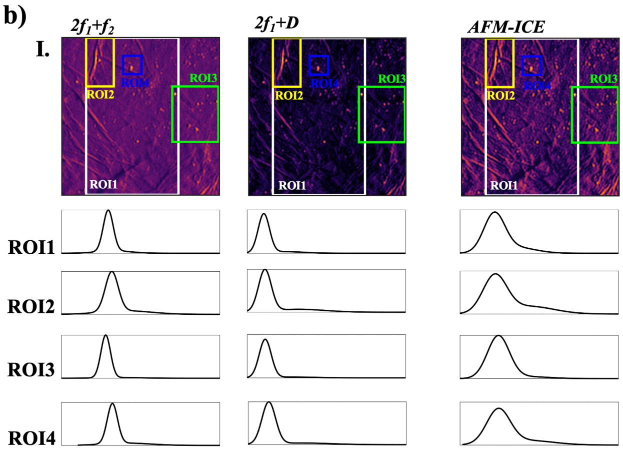

II. p-value (ANOVA)

	Original Images	+ AFM-ICE
c	0.008	0.002
STD	0.380	0.146
Entropy	0.719	0.005

III.

		$2f_1$+f_2	$2f_1$+D	AFM-ICE
ROI1	c	8.83	3.08	**8.84**
	STD	0.07	0.06	**0.11**
	Entropy	5.73	5.67	**6.64**
ROI2	c	6.98	3.14	**7.90**
	STD	0.14	0.11	**0.16**
	Entropy	6.31	6.37	**7.04**
ROI3	c	7.08	4.61	**7.60**
	STD	0.08	0.06	**0.10**
	Entropy	5.73	5.44	**6.55**
ROI4	c	5.08	3.87	**6.05**
	STD	0.12	0.10	**0.14**
	Entropy	6.05	5.97	**6.74**

Fig. S18 Deposited Au-nanoparticles on multi-layer GO-HOPG sample. a) An area of $12 \times 12\mu m$ and **b)** an area of $15 \times 15\mu$ of the sample are investigated. I. represents the two selected images from the effective cluster, the resulted image from AFM-ICE image fusion step, the ROIs considered for further analysis, and their associated histograms. II. contains the p-values obtained from one-way ANOVA test, and III. represents the calculated contrast measures for each ROI.

In: International Conference on Artificial Neural Networks, pp. 583–588 (1997). Springer

[9] Qui, K.: Kernel Principal Component Analysis (KPCA). Retrieved September 8, 2023 (2023). https://github.com/iqiukp/KPCA-MATLAB

[10] Van der Maaten, L., Hinton, G.: Visualizing data using t-sne. Journal of machine learning research **9**(11) (2008)

[11] McInnes, L., Healy, J., Melville, J.: Umap: Uniform manifold approximation and

projection for dimension reduction. arXiv preprint arXiv:1802.03426 (2018)

[12] Meehan, c., Ebrahimian, J., Moore, W., Meehan, S.: Uniform Manifold Approximation and Projection (UMAP). MATLAB Central File Exchange (2022). https://www.mathworks.com/matlabcentral/fileexchange/71902
